# Prognostic Association of Liposomal Amphotericin B Doses Above 5 mg/kg/d in Mucormycosis: A Nationwide Epidemiologic and Treatment Analysis in Japan

**DOI:** 10.1093/ofid/ofad480

**Published:** 2023-09-21

**Authors:** Masato Tashiro, Hotaka Namie, Yuya Ito, Takahiro Takazono, Hiroshi Kakeya, Yoshitsugu Miyazaki, Hiroshi Mukae, Hiroshige Mikamo, Fukuda Tomoo, Kazutoshi Shibuya, Koichi Izumikawa

**Affiliations:** Department of Infectious Diseases, Nagasaki University Graduate School of Biomedical Sciences, Nagasaki, Japan; Infection Control and Education Center, Nagasaki University Hospital, Nagasaki, Japan; Department of Infectious Diseases, Nagasaki University Graduate School of Biomedical Sciences, Nagasaki, Japan; Department of Respiratory Medicine, Nagasaki University Hospital, Nagasaki, Japan; Department of Infectious Diseases, Nagasaki University Graduate School of Biomedical Sciences, Nagasaki, Japan; Department of Respiratory Medicine, Nagasaki University Hospital, Nagasaki, Japan; Department of Infection Control Science, Graduate School of Medicine, Osaka Metropolitan University, Osaka, Japan; Department of Fungal Infection, National Institute of Infectious Disease, Tokyo, Japan; Department of Respiratory Medicine, Nagasaki University Hospital, Nagasaki, Japan; Department of Clinical Infectious Diseases, Aichi Medical University Hospital, Aichi, Japan; Department of Dermatology, Saitama Medical Center, Saitama Medical University, Saitama, Japan; Department of Pathology, Toho University Omori Medical Center, Tokyo, Japan; Department of Infectious Diseases, Nagasaki University Graduate School of Biomedical Sciences, Nagasaki, Japan; Infection Control and Education Center, Nagasaki University Hospital, Nagasaki, Japan

**Keywords:** epidemiology, liposomal amphotericin B, mucormycosis, treatment

## Abstract

**Background:**

Mucormycosis is a potentially fatal fungal infection, and there is limited information on its precise epidemiology and treatment practices, including the optimal dosage of liposomal amphotericin B.

**Methods:**

A retrospective, multicenter, nationwide analysis of 82 proven and probable cases of mucormycosis was performed. Cases between 2015 and 2022 were collected from 51 hospitals in Japan by hematologists and infectious disease specialists. The study included the epidemiology, treatment details, and association between the dose of liposomal amphotericin B and the outcome.

**Results:**

The lungs were the most commonly involved organ (70.7% of cases), and 35.4% of patients had disseminated disease. *Rhizopus* spp., *Cunninghamella* spp., and *Mucor* spp. were the most common organisms. Mortality at 4 weeks was 41.5%. The survivors had a shorter duration of neutropenia (*P* = .006) and less persistent hyperglycemia (*P* = .023). The site of infection and species of *Mucorales* had no detectable effect on survival. Survival did not differ between patients receiving liposomal amphotericin B at 5 mg/kg/d relative to those receiving >5 mg/kg/d (*P* = .625). Using Cox proportional hazards models and adjusting for confounders, the hazard ratio for the influence of >5 mg/kg/d liposomal amphotericin B on 4-week survival was 0.86 (95% CI, 0.28–2.68; *P* = .796) compared with 5 mg/kg/d.

**Conclusions:**

This study provides important insights into the precise epidemiology and treatment practices of mucormycosis. Treatment with liposomal amphotericin B at doses higher than 5 mg/kg/d did not improve outcomes relative to 5 mg/kg/d.

Mucormycosis is a rare but potentially fatal fungal infection that poses significant diagnostic and treatment challenges [1]. Individuals with underlying medical conditions such as immunodeficiency, diabetes, or coronavirus disease 2019 (COVID-19) are at increased risk for mucormycosis, which can affect various anatomical sites, including the lungs, sinuses, and skin [2]. Mucormycosis has a high mortality rate, as high as 96% in certain cases [3, 4], depending on the underlying comorbidities and site of infection. In clinical practice, there is an urgent need for comprehensive epidemiologic information on mucormycosis to facilitate timely diagnosis and prompt intervention, thereby improving treatment outcomes [5]. However, the lack of accurate epidemiologic and therapeutic data hinders effective disease management [6].

Current treatment options for mucormycosis include surgical resection and antifungal therapy [7]. Although surgical resection is the primary treatment, it is not always feasible, making antifungal therapy critical [8]. Liposomal amphotericin B is the preferred first-line antifungal agent for the treatment of mucormycosis, requiring higher doses of 5–10 mg/kg/d compared with other invasive fungal infections [9]. A particularly high dose of 10 mg/kg/d is recommended in certain cases such as brain infections or mucormycosis in solid organ transplant recipients [10, 11]. However, the optimal dose of liposomal amphotericin B for the treatment of mucormycosis remains uncertain due to the tradeoff between potentially increased efficacy and enhanced nephrotoxicity and other adverse effects associated with higher doses [12]. Therefore, it is important to evaluate the efficacy of different doses of liposomal amphotericin B to determine the optimal treatment regimen.

The aim of this nationwide observational study was to provide accurate and comprehensive epidemiologic and therapeutic information about mucormycosis in Japan. In addition, we sought to determine if administration of liposomal amphotericin B at >5 mg/kg/d to patients with mucormycosis was associated with improved outcomes. Thus, we aimed to determine the optimal dosing regimen for liposomal amphotericin B that improves therapeutic outcomes while minimizing adverse effects.

## METHODS

### Study Design and Population

The Japanese Society for Medical Mycology conducted this retrospective, multicenter, nationwide study in collaboration with the Japanese Society of Hematology and the Japanese Association for Infectious Diseases. Patient data were collected from training institutions of the Japanese Society of Hematology, certified institutions of the Japanese Association of Infectious Diseases, and members of the Japanese Society for Medical Mycology working in hospitals with >100 beds. Between January 1, 2015, and June 30, 2022, the charts of all patients in these hospitals who had been diagnosed with mucormycosis by culture, polymerase chain reaction, or pathology were analyzed. Survival data were obtained by collecting information on cases 120 days after the specimen collection date on which the first diagnosis of mucormycosis was based. Patient data were collected anonymously from each institution. The Nagasaki University Hospital Clinical Research Ethics Committee approved this study (approval number 22091210).

### Data Acquisition

Data on the various parameters were collected from individuals diagnosed with mucormycosis, including geographic regions in Japan, year of diagnosis, the rationale behind the diagnosis, site of infection, *Mucorales* genera, age, sex, underlying medical conditions, surgical interventions, and antifungal therapy. Infection sites were categorized by organ, and disseminated infections were defined as infection present in ≥2 organs. Risk factors for invasive fungal infections, including neutropenia and hematologic malignancy status, and various immunosuppressive medications for underlying diseases, such as corticosteroids, T-cell immunosuppressants, or B-cell immunosuppressants, were also evaluated [13]. Prolonged use of corticosteroids was defined as therapeutic doses ≥0.3 mg/kg/d administered for ≥3 weeks within the past 60 days, while T-cell immunosuppressants were categorized as calcineurin inhibitors, tumor necrosis factor–α blockers, lymphocyte-specific monoclonal antibodies, and immunosuppressive nucleoside analogs administered within the past 90 days. B-cell immunosuppressants were defined as Bruton's tyrosine kinase inhibitors. In addition, mucormycosis-specific comorbidities, including hyperglycemia, metabolic acidosis, intensive care, iron overload, and COVID-19, were assessed [6, 14–19].

### Epidemiology and Treatment of Mucormycosis in Japan and Characteristics of Mortality Cases

Patients were classified into proven, probable, and out-of-diagnostic definition groups according to the European Organization for Research and Treatment of Cancer and Mycoses Study Group Education and Research Consortium (EORTC/MSGERC) consensus definitions of invasive fungal disease [13]. Epidemiologic and treatment data on mucormycosis were reported for proven and probable cases, and differences in each variable were analyzed between the survivors and nonsurvivors at 4 weeks postdiagnosis. Variables included annual incidence of mucormycosis, site of infection, *Mucorales* genera, characteristics of mucormycosis patients, and characteristics of mucormycosis treatment. In addition, the geographical distribution of mucormycosis cases and *Mucorales* genera in Japan was determined. In this study, the date at which the initial specimen was collected for the diagnosis of mucormycosis was defined as day 0 for calculating the number of days of survival.

### Comparison of Liposomal Amphotericin B Doses for Prognostic Association

A comparative analysis of patients diagnosed with proven or probable mucormycosis who received liposomal amphotericin B maximum doses >5 mg/kg/d vs those who received maximum 5 mg/kg/d was performed to assess the prognostic association of liposomal amphotericin B dose on patient outcome. Patients whose maximum dose was <5 mg/kg/d or no liposomal amphotericin B were excluded, as were cases in which liposomal amphotericin B was administered for <7 days. Patient characteristics analyzed included the site of infection, causative *Mucorales* genera, age, sex, underlying disease, and neutrophil status. Treatment characteristics analyzed included timing of resection of infected lesions, antifungal agents already administered at the time of diagnosis, antifungal agents used to treat mucormycosis, and timing of initiation of liposomal amphotericin B. Outcomes analyzed were intolerance of liposomal amphotericin B leading to discontinuation, postmortem diagnosis, and probability of survival at 4, 6, and 12 weeks postdiagnosis.

### Statistical Analyses

Observed characteristics related to mucormycosis were summarized using appropriate descriptive statistics. Differences in all categorical variables were tested using the Fisher exact test or χ^2^ test. The association between liposomal amphotericin B dose and survival was evaluated using Kaplan-Meier statistics for the 5 mg/kg/d and >5 mg/kg/d liposomal amphotericin B groups. Differences were tested using the log-rank test. To control for confounding, a Cox proportional hazards model was performed, with the outcome being an interval from diagnosis to death of ≤4 weeks. The treatment variable was the maximum dose of liposomal amphotericin B (5 mg/kg/d or >5 mg/kg/d). Confounders controlled for in this analysis were age (>65 years), no recovery to >1000 neutrophils by the end of observation, and resection of the infected lesions, which clinically appear to have a clear prognostic association with the outcome of mucormycosis [14, 20, 21]. This model estimated hazard ratios (HRs) and their 95% CIs. Statistical analyses were performed using JMP 16.0 software (SAS Institute, Cary, NC, USA). All tests were 2-sided probability, and a *P* value <.05 was considered statistically significant.

## RESULTS

### Epidemiologic Characteristics of Mucormycosis in Japan

**Figure 1.**
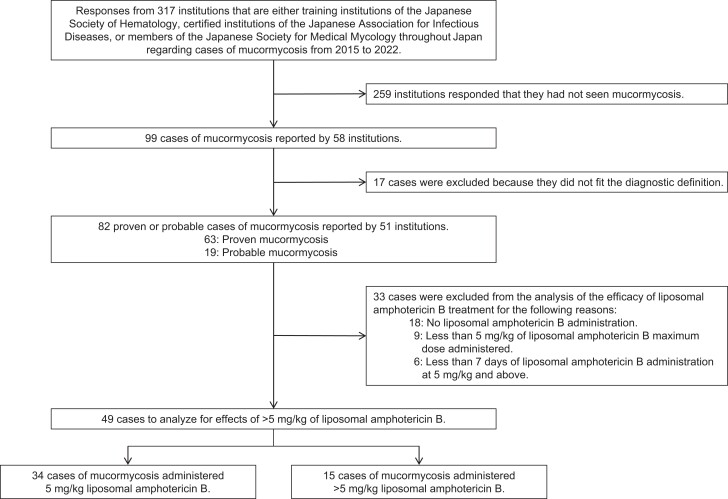
Patient selection.

We performed an analysis of 82 cases of mucormycosis from 51 institutions in Japan (Figure 1). Our results indicate no discernible disparity in the geographic distribution of cases or the causative *Mucorales* genera (*P* = .457) (Supplementary Table 1). Furthermore, we did not observe a significant increase in the incidence of cases after the COVID-19 pandemic (Table 1). The most common organ involved was the lungs, followed by the paranasal sinuses and the skin. Disseminated infection occurred in 35.4% of cases. Identification of *Mucorales* by culture was reported in 45.1% of cases. *Rhizopus* spp., *Cunninghamella* spp., and *Mucor* spp. were the most common *Mucorales* genera. No significant differences were observed in the distribution of infected organs or *Mucorales* genera between survivors and nonsurvivors. Notably, blood cultures showed a significantly higher detection of *Mucor* spp. compared with other organs (*P* < .001), as shown in Supplementary Table 2. In addition, Supplementary Table 2 contains the underlying data used to establish proven and probable determinations for the patients in this study.

**Table 1.** Comparison of the Number of Cases of Mucormycosis per Year in Japan, the Site of Infection, and the Causative Organism in Survivors and Nonsurvivors

Variable	All n = 82		Survivors n = 48		Nonsurvivors^a^ n = 34		*P* Value^b^
Year, No. (%)							
2015	11	(13.4)	4	(36.4)	7	(63.6)	.432
2016	14	(17.1)	9	(64.3)	5	(35.7)	
2017	9	(11.0)	5	(55.6)	4	(44.4)	
2018	7	(8.5)	5	(71.4)	2	(28.6)	
2019	15	(18.3)	10	(66.7)	5	(33.3)	
2020	12	(14.6)	8	(66.7)	4	(33.3)	
2021	9	(11.0)	6	(66.7)	3	(33.3)	
2022	5	(6.1)	1	(20.0)	4	(80.0)	
Site of infections, No. (%)							
Pulmonary	58	(70.7)	36	(62.1)	22	(37.9)	.335
Sinus	16	(19.5)	10	(62.5)	6	(37.5)	.784
Skin	13	(15.9)	7	(53.8)	6	(46.2)	.765
Cerebral	9	(11.0)	4	(44.4)	5	(55.6)	.478
Blood	8	(9.8)	6	(75.0)	2	(25.0)	.459
Gastrointestinal tissue	7	(8.5)	2	(28.6)	5	(71.4)	.120
Other organs^c^	14	(17.1)	6	(42.9)	8	(57.1)	.239
Disseminated^d^	29	(35.4)	14	(48.3)	15	(51.7)	.241
Organism, No. (%)^e^							
*Rhizopus* spp.	17	(20.7)	10	(58.8)	7	(41.2)	1.000
*Cunninghamella* spp.	15	(18.3)	8	(53.3)	7	(46.7)	.774
*Mucor* spp.	15	(18.3)	8	(53.3)	7	(46.7)	.774
*Rhizomucor* spp.	7	(8.5)	2	(28.6)	5	(71.4)	.120
*Lichtheimia* spp.	1	(1.2)	1	(100.0)	0	(0.0)	1.000
Genus not identified	28	(34.1)	19	(67.9)	9	(32.1)	.246

The number of infection sites and causative organisms includes duplicates.

a Nonsurvivors within 4 weeks of diagnosis. Mortality rates at each time point were 41.5% (34/82) at 4 weeks, 45.1% (37/82) at 6 weeks, and 52.4% (43/82) at 12 weeks after diagnosis. The definition of “time of diagnosis” here refers to the date when the specimen with *Mucorales* was collected.

b The *P* value represents statistical differences between survivors and nonsurvivors. In cases where the variable consists of items without duplicates, we conducted chi-square tests using a table of the number of items multiplied by 2, rather than a 2 × 2 contingency table for each item. For example, the variable “year” presents the results of a chi-square test on an 8 × 2 contingency table (8 categories from 2015 to 2022, 2 categories for survivors vs nonsurvivors), and therefore only 1 *P* value is indicated for “year.”

c Other organs included the thyroid, bronchus, pleura, heart, spine, liver, pancreas, spleen, kidney, bladder, and retroperitoneum.

d Cases with confirmed infection of 2 or more organs were defined as disseminated.

e Of the 55 strains identified, 37 (67.3%) organisms were identified by culture morphology, 27 (49.1%) organisms by genetic identification, and 9 (16.4%) organisms by time-of-flight mass spectrometry.

### Patient Backgrounds and Treatment Characteristics in Fatal Cases of Mucormycosis

Mucormycosis was diagnosed in all age groups. No significant difference in mortality was observed among the different age groups (*P* = .880) (Table 2), but the relatively small number of pediatric cases limits the statistical power of this analysis. Host factors as defined by EORTC/MSGERC that are associated with increased risk of developing mucormycosis were identified in 84.1% of cases. Hematologic malignancy was present in 69.5% of cases and hyperglycemia in 14.6%. Of the 12 hyperglycemic patients, 3 were accompanied by metabolic acidosis. Only 2 cases occurred in patients who were being treated for COVID-19. The nonsurvivors had a longer duration of neutropenia (*P* = .006), persistent hyperglycemia (*P* = .023), and a higher incidence of the need for intensive care (*P* < .001) (Table 2). However, there were no significant differences in other patient characteristics, including underlying diseases, between the survivors and nonsurvivors. Supplementary Table 3 shows the background characteristics of the patients for each infected organ.

**Table 2.** Comparison of Characteristics of Mucormycosis Patients in Survivors and Nonsurvivors

Variable	All n = 82		Survivors n = 48		Nonsurvivors^a^ n = 34		*P* Value^b^
Age, No. (%)							
0 y	4	(4.9)	3	(75.0)	1	(25.0)	.880
1–12 y	6	(7.3)	3	(50.0)	3	(50.0)	
13–17 y	7	(8.5)	5	(71.4)	2	(28.6)	
18–64 y	33	(40.2)	19	(57.6)	14	(42.4)	
≥65 y	32	(39.0)	18	(56.3)	14	(43.8)	
Male, No. (%)	55	(67.1)	31	(56.4)	24	(43.6)	.638
Underlying condition at the time of diagnosis, No. (%)^c^							
Neutrophil <500/µL for ≥10 d	24	(29.3)	8	(33.3)	16	(66.7)	.006
Neutrophil <500/µL for ≥30 d	17	(20.7)	6	(35.3)	11	(64.7)	.051
Hematologic malignancy	57	(69.5)	34	(59.6)	23	(40.4)	.810
Allogeneic stem cell transplant	28	(34.1)	16	(57.1)	12	(42.9)	1.000
Acute graft-vs-host disease grade I	4	(4.9)	4	(100.0)	0	(0.0)	.138
Acute graft-vs-host disease grade II	6	(7.3)	4	(66.7)	2	(33.3)	1.000
Acute graft-vs-host disease grade III	0	(0.0)	0	-	0	-	-
Acute graft-vs-host disease grade IV	3	(3.7)	1	(33.3)	2	(66.7)	.567
Solid organ transplant	3	(3.7)	0	(0.0)	3	(100.0)	.068
Prolonged use of corticosteroids^d^	38	(46.3)	18	(47.4)	20	(52.6)	.073
T-cell immunosuppressants^e^	28	(34.1)	12	(42.9)	16	(57.1)	.058
B-cell immunosuppressants^f^	5	(6.1)	2	(40.0)	3	(60.0)	.644
Persistent hyperglycemia	12	(14.6)	3	(25.0)	9	(75.0)	.023
Metabolic acidosis	6	(7.3)	2	(33.3)	4	(66.7)	.226
Undergoing treatment in the intensive care unit	27	(32.9)	6	(22.2)	21	(77.8)	<.001
Undergoing treatment for iron overload	8	(9.8)	4	(50.0)	4	(50.0)	.713
Undergoing treatment for COVID-19	2	(2.4)	0	(0.0)	2	(100.0)	.169
Neutrophil recovered to >1000/µL, No. (%)^g^							
Already ≥1000/µL at diagnosis	52	(63.4)	38	(73.1)	14	(26.9)	<.001
Recovered within 7 d of diagnosis	2	(2.4)	2	(100.0)	0	(0.0)	
Recovered within 8–14 d of diagnosis	4	(4.9)	2	(50.0)	2	(50.0)	
Recovered ≥15 d after diagnosis	2	(2.4)	2	(100.0)	0	(0.0)	
No recovery to >1000 neutrophils by the end of observation	20	(24.4)	2	(10.0)	18	(90.0)	

Abbreviation: COVID-19, coronavirus disease 2019.

a Nonsurvivors within 4 weeks of diagnosis. Mortality rates at each time point were 41.5% (34/82) at 4 weeks, 45.1% (37/82) at 6 weeks, and 52.4% (43/82) at 12 weeks after diagnosis. The definition of “time of diagnosis” here refers to the date when the specimen with *Mucorales* was collected.

b The *P* value represents statistical differences between survivors and nonsurvivors. In cases where the variable consists of items without duplicates, we conducted chi-square tests using a table of the number of items multiplied by 2, rather than a 2 × 2 contingency table for each item.

c There were no patients with inherited severe immunodeficiency or HIV infection.

d Prolonged use of corticosteroids at a therapeutic dose of ≥0.3 mg/kg/d corticosteroids for ≥3 weeks in the past 60 days.

e T-cell immunosuppressants, such as calcineurin inhibitors, tumor necrosis factor–α blockers, lymphocyte-specific monoclonal antibodies, and immunosuppressive nucleoside analogs, during the past 90 days.

f B-cell immunosuppressants, such as Bruton's tyrosine kinase inhibitors, for example, ibrutinib.

g Neutrophil status data were not available in 2 cases.

Surgical resection of the fungal lesions was performed in 34.1% of cases, and there were more surgical resections in the patients who survived (*P* < .001) (Table 3). Several antifungal medications were administered before confirmation of the diagnosis. Liposomal amphotericin B was administered as the sole antifungal agent in 58.5% of cases, while 19.3% received this drug in combination with other antifungals. However, the types of antifungals used and the use of combination therapy had no effect on survival (*P* = .244). The maximum dose of liposomal amphotericin B used varied from <5 mg/kg/d to >10 mg/kg/d, but there was no difference in the doses administered between survivors and nonsurvivors (*P* = .570). Of the 64 patients treated with liposomal amphotericin B, 15 (23.4%) required dose escalation due to poor response to treatment, and 6 (9.4%) discontinued treatment due to intolerance to liposomal amphotericin B. The median duration of treatment with liposomal amphotericin B ≥5 mg/kg/d in the survival group (interquartile range) was 36.5 (0–70) days. The timing of initiation of liposomal amphotericin B at a dose of ≥5 mg/kg/d was not significantly different between survivors and nonsurvivors (*P* = .237) (Table 3). Treatment details by infected organ are summarized in Supplementary Table 4.

**Table 3.** Comparison of Characteristics of Mucormycosis Treatment in Survivors and Nonsurvivors

Variable	All n = 82		Survivors n = 48		Nonsurvivors^a^ n = 34		*P* Value^b^
Resection of infected lesions, No. (%)							
Within 3 d of diagnosis	20	(24.4)	19	(95.0)	1	(5.0)	<.001
Within 4–7 d of diagnosis	2	(2.4)	2	(100.0)	0	(0.0)	
Within 8–14 d of diagnosis	2	(2.4)	2	(100.0)	0	(0.0)	
After 15 d of diagnosis	4	(4.9)	4	(100.0)	0	(0.0)	
No resection	54	(65.9)	21	(38.9)	33	(61.1)	
Antifungal drugs already used at diagnosis, No. (%)^c^							
Fluconazole	4	(4.9)	2	(50.0)	2	(50.0)	1.000
Itraconazole	6	(7.3)	4	(66.7)	2	(33.3)	1.000
Voriconazole	17	(20.7)	8	(47.1)	9	(52.9)	.407
Posaconazole	3	(3.7)	1	(33.3)	2	(66.7)	.567
Micafungin	12	(14.6)	8	(66.7)	4	(33.3)	.753
Caspofungin	10	(12.2)	3	(30.0)	7	(70.0)	.084
Liposomal amphotericin B	23	(28.0)	17	(73.9)	6	(26.1)	.088
Antifungal drugs administered as a treatment for mucormycosis, No. (%)^c^							
Liposomal amphotericin B alone	48	(58.5)	31	(64.6)	17	(35.4)	.244
Posaconazole alone^d^	3	(3.7)	2	(66.7)	1	(33.3)	
Liposomal amphotericin B+ posaconazole	2	(2.4)	2	(100.0)	0	(0.0)	
Liposomal amphotericin B+ micafungin	2	(2.4)	2	(100.0)	0	(0.0)	
Liposomal amphotericin B+ caspofungin	6	(7.3)	3	(50.0)	3	(50.0)	
Liposomal amphotericin B+ others^e^	6	(7.3)	3	(50.0)	3	(50.0)	
No effective antifungal administration^f^	15	(18.3)	5	(33.3)	10	(66.7)	
Maximum dose of liposomal amphotericin B, No. (%)							
No administration	18	(22.0)	7	(38.9)	11	(61.1)	.570
<5 mg/kg/d	9	(11.0)	6	(66.7)	3	(33.3)	
5 mg/kg/d	39	(47.6)	24	(61.5)	15	(38.5)	
6 mg/kg/d	4	(4.9)	2	(50.0)	2	(50.0)	
7 mg/kg/d	1	(1.2)	1	(100.0)	0	(0.0)	
8 mg/kg/d	3	(3.7)	2	(66.7)	1	(33.3)	
9 mg/kg/d	2	(2.4)	1	(50.0)	1	(50.0)	
≥10 mg/kg/d	6	(7.3)	5	(83.3)	1	(16.7)	
Start liposomal amphotericin B administration at ≥5 mg/kg/d, No. (%)							
Before diagnosis	20	(24.4)	14	(70.0)	6	(30.0)	.237
Within 3 d of diagnosis	21	(25.6)	10	(47.6)	11	(52.4)	
Within 4–7 d of diagnosis	8	(9.8)	6	(75.0)	2	(25.0)	
Within 8–14 d of diagnosis	7	(8.5)	6	(85.7)	1	(14.3)	
After 15 d of diagnosis	2	(2.4)	1	(50.0)	1	(50.0)	
No administration of liposomal amphotericin B at ≥5 mg/kg/d	24	(29.3)	11	(45.8)	13	(54.2)	

a Nonsurvivors within 4 weeks of diagnosis. Mortality rates at each time point were 41.5% (34/82) at 4 weeks, 45.1% (37/82) at 6 weeks, and 52.4% (43/82) at 12 weeks after diagnosis. The definition of “time of diagnosis” here refers to the date when the specimen with *Mucorales* was collected.

b The *P* value represents statistical differences between survivors and nonsurvivors. In cases where the variable consists of items without duplicates, we conducted chi-square tests using a table of the number of items multiplied by 2, rather than a 2 × 2 contingency table for each item.

c Isavuconazole was not approved in Japan at the time of data collection.

d One case of a combination of posaconazole and caspofungin was included.

e Other antifungals used in combination were fluconazole and itraconazole in 1 case each and voriconazole in 4 cases.

f Patients not receiving liposomal amphotericin B or posaconazole.

Mortality rates at each time point were 41.5% (34/82) at 4 weeks, 45.1% (37/82) at 6 weeks, and 52.4% (43/82) at 12 weeks after diagnosis. The number of patients diagnosed at postmortem examination was 9 (10.8%). No prognostic differences were observed based on specific organ involvement (Supplementary Table 5).

### Effects of Liposomal Amphotericin B at 5 mg/kg/d and >5 mg/kg/d on Outcome

**Figure 2.**
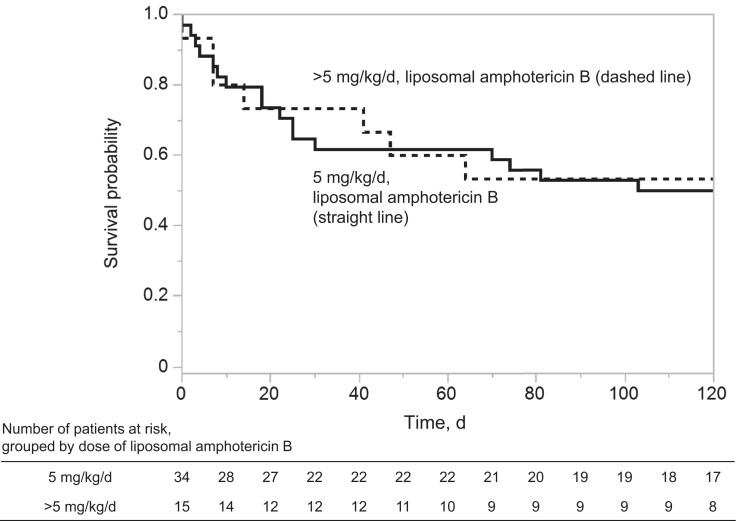
Kaplan-Meier estimates of survival in the 2 groups of mucormycosis treated with different doses of liposomal amphotericin B. Cases with a total duration of liposomal amphotericin B administration of <7 days were excluded from the analysis. However, because day 0 is defined as the day of diagnosis and includes cases in which liposomal amphotericin B was initiated before day 0, there are cases in which death occurred within 7 days.

The survival of patients with mucormycosis in 34 patients treated with 5 mg/kg/d of liposomal amphotericin B (5 mg/kg/d group) was compared with that of 15 patients who were treated with a dose >5 mg/kg/d (>5 mg/kg/d group) (Figure 1). No significant differences were observed between the 2 groups in terms of infected organs, *Mucorales* genera, or patient background except for brain infection, which occurred only in the patients who received >5 mg/kg/d (Table 4). There were no significant differences between the 2 groups in terms of surgical resection of the infected site, antifungal administration before diagnosis, concomitant use of antifungal agents, or timing of liposomal amphotericin B administration (Table 5). Survival at 4, 6, and 12 weeks did not differ between the 2 groups (*P* = .625, *P* = .769, and *P* = .981, respectively) (Table 6, Figure 2). Using Cox proportional hazards modeling that included confounders such as age, recovery of neutrophil count to >1000, and surgical resection, which may influence the prognosis of mucormycosis, the hazard ratio for the effect of >5 mg/kg/d liposomal amphotericin B on prognosis at 4 weeks was 0.86 (95% CI, 0.28–2.68; *P* = .796) compared with 5 mg/kg/d, and no superiority of >5 mg/kg/d over 5 mg/kg/d was demonstrated (Supplementary Table 6).

**Table 4.** Comparison of Characteristics of Mucormycosis Patients Treated With Liposomal Amphotericin B at 5 mg/kg/d and Mucormycosis Patients Treated With >5 mg/kg/d

Variable	5 mg/kg/d Liposomal Amphotericin B n = 34		>5 mg/kg/d Liposomal Amphotericin B^a^ n = 15		*P* Value
Site of infections^b^					
Pulmonary	24	(70.6)	9	(60.0)	.520
Sinus	6	(17.6)	4	(26.7)	.470
Skin	4	(11.8)	4	(26.7)	.227
Cerebral	0	(0.0)	5	(33.3)	.002
Blood	3	(8.8)	3	(20.0)	.353
Gastrointestinal tissue	5	(14.7)	0	(0.0)	.306
Other organs^c^	4	(11.8)	2	(13.3)	1.000
Disseminated^d^	10	(29.4)	7	(46.7)	.331
*Mucorales*, No. (%)					
*Rhizopus* spp.	8	(23.5)	5	(33.3)	.500
*Cunninghamella* spp.	5	(14.7)	2	(13.3)	1.000
*Mucor* spp.	6	(17.6)	3	(20.0)	1.000
*Rhizomucor* spp.	5	(14.7)	0	(0.0)	.306
*Lichtheimia* spp.	1	(2.9)	0	(0.0)	1.000
Genus not identified	9	(26.5)	5	(33.3)	.735
Age, No. (%)					
0 y	4	(11.8)	0	(0.0)	.373
1–12 y	3	(8.8)	2	(13.3)	
13–17 y	4	(11.8)	0	(0.0)	
18–64 y	16	(47.1)	9	(60.0)	
≥65 y	7	(20.6)	4	(26.7)	
Male, No. (%)	25	(73.5)	9	(60.0)	.502
Underlying condition at the time of diagnosis, No. (%)^e^					
Severe neutropenia for >10 d	12	(35.3)	5	(33.3)	1.000
Severe neutropenia for >30 d	10	(29.4)	2	(13.3)	.298
Hematologic malignancy	29	(85.3)	11	(73.3)	.427
Allogeneic stem cell transplant	16	(47.1)	7	(46.7)	1.000
Acute graft-vs-host disease grade I	1	(2.9)	2	(13.3)	.218
Acute graft-vs-host disease grade II	6	(17.6)	0	(0.0)	.159
Acute graft-vs-host disease grade III	0	(0.0)	0	(0.0)	-
Acute graft-vs-host disease grade IV	1	(2.9)	0	(0.0)	1.000
Solid organ transplant	0	(0.0)	0	(0.0)	-
Prolonged use of corticosteroids^f^	16	(47.1)	8	(53.3)	.762
T-cell immunosuppressants^g^	16	(47.1)	4	(26.7)	.221
B-cell immunosuppressants^h^	4	(11.8)	0	(0.0)	.298
Persistent hyperglycemia	3	(8.8)	4	(26.7)	.179
Metabolic acidosis	2	(5.9)	2	(13.3)	.576
Undergoing treatment in the intensive care unit	11	(32.4)	4	(26.7)	.750
Undergoing treatment for iron overload	3	(8.8)	1	(6.7)	1.000
Undergoing treatment for COVID-19	1	(2.9)	0	(0.0)	1.000
Neutrophil recovered to >1000/µL, No. (%)					
Already >1000/µL at diagnosis	22	(64.7)	8	(53.3)	.583
Recovered within 7 d of diagnosis	1	(2.9)	0	(0.0)	
Recovered within 8–14 d of diagnosis	1	(2.9)	2	(13.3)	
Recovered ≥15 d after diagnosis	1	(2.9)	1	(6.7)	
No recovery to >1000 neutrophils by the end of observation	9	(26.5)	4	(26.7)	

The number of infection sites and causative organisms includes duplicates. The definition of “time of diagnosis” here refers to the date on which the specimen with *Mucorales* was taken.

Abbreviation: COVID-19, coronavirus disease 2019.

a Liposomal amphotericin B was administered in 3 cases at 6 mg/kg/d, 1 case at 7 mg/kg/d, 3 cases at 8 mg/kg/d, 2 cases at 9 mg/kg/d, and 6 cases at ≥10 mg/kg/d.

b No cases of orbital lesions were identified.

c Other organs included the bronchus, liver, spleen, bladder, and retroperitoneum.

d Cases with confirmed infection of 2 or more organs were defined as disseminated.

e There were no patients with inherited severe immunodeficiency or HIV infection.

f Prolonged use of corticosteroids at a therapeutic dose of ≥0.3 mg/kg/d corticosteroids for ≥3 weeks in the past 60 days.

g T-cell immunosuppressants, such as calcineurin inhibitors, tumor necrosis factor–α blockers, lymphocyte-specific monoclonal antibodies, and immunosuppressive nucleoside analogs, during the past 90 days.

h B-cell immunosuppressants, such as Bruton's tyrosine kinase inhibitors, for example, ibrutinib.

**Table 5.** Comparison of Treatment Characteristics of Mucormycosis Patients Treated With Liposomal Amphotericin B at 5 mg/kg/d and Mucormycosis Patients Treated With >5 mg/kg/d

Variable	5 mg/kg/d Liposomal Amphotericin B n = 34		>5 mg/kg/d Liposomal Amphotericin B^a^ n = 15		*P* Value
Resection of infected lesions, No. (%)					
Within 3 d of diagnosis	10	(29.4)	4	(26.7)	.811
Within 4–7 d of diagnosis	1	(2.9)	0	(0.0)	
Within 8–14 d of diagnosis	1	(2.9)	1	(6.7)	
After 15 d of diagnosis	2	(5.9)	2	(13.3)	
No resection	20	(58.8)	8	(53.3)	
Antifungal drugs already used at diagnosis, No. (%)					
Fluconazole	2	(5.9)	0	(0.0)	1.000
Itraconazole	2	(5.9)	0	(0.0)	1.000
Voriconazole	7	(20.6)	3	(20.0)	1.000
Posaconazole	1	(2.9)	0	(0.0)	1.000
Micafungin	5	(14.7)	2	(13.3)	1.000
Caspofungin	5	(14.7)	2	(13.3)	1.000
Liposomal amphotericin B	13	(38.2)	8	(53.3)	.363
Antifungal drugs administered as a treatment for mucormycosis, No. (%)					
Liposomal amphotericin B alone	27	(79.4)	9	(60.0)	.072
Liposomal amphotericin B+ posaconazole	1	(2.9)	1	(6.7)	
Liposomal amphotericin B+ micafungin	0	(0.0)	1	(6.7)	
Liposomal amphotericin B+ caspofungin	2	(5.9)	4	(26.7)	
Liposomal amphotericin B+ other^b^	4	(11.8)	0	(0.0)	
Start liposomal amphotericin B administration at ≥5 mg/kg/d, No. (%)					
Before diagnosis	10	(29.4)	7	(46.7)	.293
Within 3 d of diagnosis	11	(32.4)	6	(40.0)	
Within 4–7 d of diagnosis	6	(17.6)	0	(0.0)	
Within 8–14 d of diagnosis	6	(17.6)	1	(6.7)	
≥15 d after diagnosis	1	(2.9)	1	(6.7)	

The definition of “time of diagnosis” here refers to the date on which the specimen with *Mucorales* was taken.

a Liposomal amphotericin B was administered in 3 cases at 6 mg/kg/d, 1 case at 7 mg/kg/d, 3 cases at 8 mg/kg/d, 2 cases at 9 mg/kg/d, and 6 cases at ≥10 mg/kg/d.

b Other antifungals used in combination were itraconazole in 1 case and voriconazole in 3 cases.

**Table 6.** Comparison of Outcomes of Mucormycosis Patients Treated With Liposomal Amphotericin B at 5 mg/kg/d and Those Treated With >5 mg/kg/d

Variable	5 mg/kg/d Liposomal Amphotericin B n = 34		>5 mg/kg/d Liposomal Amphotericin B^a^ n = 15		*P* Value
Treatment discontinuation due to liposomal amphotericin B intolerance, No. (%)	3	(8.8)	1	(6.7)	1.000
Diagnosed at postmortem examination, No. (%)	1	(2.9)	1	(6.7)	.523
Survival probabilities (95% CI),^b^%					
At 4 wk	64.7	(47.6−78.7)	73.3	(46.7−89.6)	.625
At 6 wk	61.8	(44.7−76.3)	66.7	(40.6−85.4)	.769
At 12 wk	52.9	(36.5−68.8)	53.3	(29.3−75.9)	.981

a Liposomal amphotericin B was administered in 3 cases at 6 mg/kg/d, 1 case at 7 mg/kg/d, 3 cases at 8 mg/kg/d, 2 cases at 9 mg/kg/d, and 6 cases at ≥10 mg/kg/d.

b Survival probabilities were estimated using the Kaplan-Meier method.

## DISCUSSION

This study elucidated the precise epidemiology and treatment outcomes of patients with mucormycosis across the adult and pediatric spectrum. The strength of this study lies in the analysis of mucormycosis cases with strict and precise definitions, as shown in Supplementary Table 2. Data were collected from 51 institutions across Japan without geographical or institutional bias, with an average of 1.6 patients per institution. The value of this study lies in the inclusion of a strict case definition and an unbiased patient cohort, both of which are essential for epidemiologic and treatment analyses. Moreover, the effect of liposomal amphotericin B at doses >5 mg/kg/d on prognosis was directly compared with 5 mg/kg/d, providing important insights into the optimal dose for effective treatment in this patient population.

This study showed that the mortality rate for mucormycosis managed by hematologists and infectious disease specialists, regardless of the organ involved, remains substantial at 41.5% at 4 weeks after diagnosis. Furthermore, the stable mortality rate from 2015 to the present suggests a lack of improvement in treatment strategies for mucormycosis. Contrary to previous reports, the prognosis of mucormycosis treated by hematologists and infectious disease specialists did not vary significantly by organ involved [4, 14]. This finding may be influenced by disseminated infection, which occurred in 35.4% of cases. Due to the characteristics of the study population, the main analysis was performed without stratifying by infected organ type. The influence of patient background on the prognosis of mucormycosis was also an interesting finding. It highlights the significant influence of host factors on the development of mucormycosis, as 84.1% of cases in this study had host factors as defined by EORTC/MSGERC. However, the types of host factors that affect mortality in mucormycosis are limited, and this study only demonstrated the potential association of prolonged neutropenia and persistent hyperglycemia on mortality. In addition, mucormycosis affected people of all ages, and mortality rates did not differ by age group. These findings suggest that host immune status, particularly recovery from neutropenia, may have a greater impact on prognosis than age and other underlying conditions.

Our study also shed light on the epidemiology of mucormycosis in Japan. Although there has been a worldwide increase in the incidence of COVID-19-associated mucormycosis [5, 22], this infection appears to have had a negligible impact on the epidemiology of mucormycosis in Japan. In this study, we confirmed that *Rhizopus* spp. was the most common causative microorganism of mucormycosis, as in other countries [1, 6, 9, 15]; however, in contrast to previous reports, we also found that *Cunninghamella* spp. was as common as *Mucor* spp. It has been reported that *Cunninghamella* spp. are frequently isolated from pulmonary and disseminated lesions [6]. Our focus on hematologists and infectious disease specialists in this study may have contributed to the high incidence of pulmonary and disseminated mucormycosis and thus to a higher incidence of *Cunninghamella* spp. than previously reported. It has also been reported that mucormycosis caused by *Cunninghamella* spp. has a higher mortality rate than infections caused by other genera [4, 6], although this was not observed in this study. This difference may be because patients infected with *Cunninghamella* spp. were more likely to have pulmonary or disseminated mucormycosis and were more likely to die because of their complex clinical history. This study also yielded interesting results regarding the importance of blood cultures in mucormycosis. Our results showed that 9.8% of mucormycosis patients managed by hematologists and infectious disease specialists had positive blood cultures, particularly those with *Mucor* spp. Adding blood culture to the diagnostic protocol for mucormycosis patients with host factors and *Mucor* spp. may be beneficial, particularly in the case of disseminated mucormycosis.

One of the epidemiologic features of mucormycosis in Japan is the low prevalence of hyperglycemia (14.6%) compared with reports from other countries. The prevalence of diabetes mellitus as an underlying disease in patients with mucormycosis varies widely in different regions of the world. It ranges from 44% to 75% in India and Iran, 52% in the United States, 27% in Australia, and 17% to 23% in Europe [23]. Our findings showed the lowest proportion compared with these reports. The reported prevalence of type 2 diabetes per 100 000 people in 2017 was 6059 worldwide, 6737 in Japan, 4770 in India, and 8911 in the United States, and the lowest in Europe was 6843 in France [24]. Thus, it can be concluded that the prevalence of diabetes in Japan is not low. Furthermore, this study identified only 3 cases (3.7%) of suspected diabetic ketoacidosis, and there are no direct comparative reports on the incidence of diabetic ketoacidosis in Japan and other countries, so the reasons for this disparity are unknown.

Treatment-related findings were observed, with surgical resection being frequently used in the survival group. While the design of the present study precludes a definitive determination of causality between surgery and prognosis, it remains a pivotal observation that the 4-week survival rate in patients without surgery was only 38.9%, in stark contrast to the 95% survival rate demonstrated in patients who underwent surgical resection within 3 days of diagnosis. Combination of liposomal amphotericin B with other antifungal agents did not show a detectable synergistic effect. There was no significant difference in the timing of initiation of treatment with ≥5 mg/kg/d of liposomal amphotericin B between the survivors and nonsurvivors.

Our study evaluated the clinical outcomes of liposomal amphotericin B administered at different doses for mucormycosis. We found no significant differences in outcome between the 5 mg/kg/d and >5 mg/kg/d groups. Even after adjustment for some limited confounders, no survival benefit was observed with doses >5 mg/kg/d. The results support the use of 5 mg/kg/d as an appropriate starting dose in the treatment of mucormycosis, although it should be noted that this study cannot exclude the possibility that >5 mg/kg/d was used in patients with more severe disease. Clinicians would benefit from evidence supporting the use of 5 mg/kg/d because very high doses (eg, 10 mg/kg/d) have been associated with increased nephrotoxicity and hypokalemia [12]. Because data on renal function and potassium were not collected in this study, it was not possible to assess the incidence of nephrotoxicity or hypokalemia in the high-dose group. It is important to note that current treatment guidelines recommend a dose of 10 mg/kg/d in cases of brain infection and solid organ transplantation [7]. In our study, all 5 cases of brain infection were treated with doses >5 mg/kg/d and none with 5 mg/kg/d. In addition, all 3 patients with solid organ transplantation died, making it impossible to determine the efficacy of different doses of liposomal amphotericin B in such cases.

The limitations of this study should be considered when interpreting the results. First, mild cases of mucormycosis with primarily superficial lesions may have been underrepresented because data were not collected from specialists such as otolaryngologists and dermatologists. Therefore, the epidemiologic and treatment outcomes of this study may only represent the characteristics of mucormycosis seen by hematologists and infectious disease specialists. Second, nonresponse bias may exist because not all hospitals in Japan were studied. Nevertheless, responses were obtained from 317 institutions—representing 37.8% of the total 838 target institutions, consisting of training institutions of the Japanese Society of Hematology, certified institutions of the Japanese Association for Infectious Diseases, and some of the members of the Japanese Society for Medical Mycology—and thus were likely to provide accurate epidemiologic data. Third, the impact of isavuconazole on mucormycosis was not considered in this study because isavuconazole was approved in Japan in December 2022. Fourth, the end point in our study did not include nephrotoxicity. At a higher dose of 10 mg/kg/d, it has been reported that 40% of patients experienced dose reduction or discontinuation due to nephrotoxicity [25], making the data on nephrotoxicity at high doses important when considering liposomal amphotericin B dosing. We evaluated discontinuation due to adverse events as an alternative end point for nephrotoxicity in our study (Table 6). Within the group receiving doses >5 mg/kg/d, only 1 case (6.7%) resulted in discontinuation due to adverse events, which appears to be lower than the prospective study data for the 10-mg/kg/d dose [25]. The limitation of 10 mg/kg/d cases to only 6 cases (40%) within the >5 mg/kg/d group in this study may have influenced the differences from previous data. Finally, the lack of statistical power of this study design is evident when evaluating the differential effects of the high and standard doses of liposomal amphotericin B on outcomes. In a prospective pilot study by Lanternier et al., the 4-week survival rate of mucormycosis patients treated with high-dose (10 mg/kg/d) liposomal amphotericin B was 79% [25], whereas in another prospective study, the DEFEAT study by Spellberg et al., the 4-week survival rate of mucormycosis patients treated with the standard dose was 70% [26]. Using these data and setting the alpha level at .05 and the power at 80%, the required sample size is calculated to be 369 cases per group. In our study, the 5 mg/kg/d group consisted of 34 cases, while the >5 mg/kg/d group consisted of 15 cases. Due to the insufficient number of cases to effectively detect differences in outcomes, there remains the possibility that our study may not detect significant differences in outcomes between the 2 groups. In addition, unadjusted confounding factors, such as doses >5 mg/kg/d in patients with severe disease or renal function influencing dosage decisions, may have influenced the results of this retrospective observational study evaluating the prognostic association of antifungal therapy, despite adjustment for some confounding factors.

In conclusion, we have successfully elucidated the precise epidemiology and treatment outcomes of mucormycosis managed by hematologists and infectious disease specialists in Japan. In this study, we found that there was no difference in the outcome of mucormycosis in patients who received liposomal amphotericin B at 5 mg/kg/d vs >5 mg/kg/d. As the evidence for the treatment of mucormycosis is limited mainly to observational and retrospective studies, additional clinical trials are needed to improve the prognosis of this disease. We believe that the results of our study will provide insight into the management of current mucormycosis cases and provide a foundation for future research.

## Supplementary Material

ofad480_Supplementary_Data

## Supplementary Data

Supplementary materials are available at *Open Forum Infectious Diseases* online. Consisting of data provided by the authors to benefit the reader, the posted materials are not copyedited and are the sole responsibility of the authors, so questions or comments should be addressed to the corresponding author.

## Notes

***Financial support.*** This work was supported by the Ministry of Health, Labour and Welfare (MHLW) Research on Emerging and Re-emerging Infectious Diseases and Immunization (Program Grant Number JPMH21HA2011).

***Patient consent.*** Patient data were collected anonymously from each institution. The Nagasaki University Hospital Clinical Research Ethics Committee approved this study (approval number 22091210). The Ethics Committee determined that individual informed consent was not required for this study.
