# Supplementary material for: Prognostic Association of Liposomal Amphotericin B Doses Above 5 mg/kg/d in Mucormycosis: A Nationwide Epidemiologic and Treatment Analysis in Japan
Source: Open Forum Infect Dis. 2023 Sep 21;10(10):ofad480. doi: 10.1093/ofid/ofad480 (PMC10552064; doi:10.1093/ofid/ofad480)
Supplement: ofad480_Supplementary_Data [file ofad480_supplementary_data.pdf]

Table S1. The number of responding institutions, mucormycosis cases, and distribution of *Mucorales* in each prefectural region of Japan.

Prefectural Regions in Japan [a]	Population in 2022 [b]	Number of responding institutions n = 317	Number of all cases n = 99	Number of proven or probable cases n = 82	*Rhizopus* spp. n = 17	*Cunninghamella* spp. n = 15	*Mucor* spp. n = 15	*Rhizomucor* spp. n = 7	*Lichtheimia* spp. n = 1	Species not identified n = 28
Hokkaido	5,183,687	13	2	2	0	0	1	0	0	1
Aomori	1,243,081	0	0	0	0	0	0	0	0	0
Iwate	1,206,479	2	0	0	0	0	0	0	0	0
Miyagi	2,268,355	4	3	2	1	1	0	0	0	0
Akita	956,836	3	0	0	0	0	0	0	0	0
Yamagata	1,056,682	4	2	2	0	2	1	0	0	0
Fukushima	1,841,244	3	0	0	0	0	0	0	0	0
Ibaraki	2,890,377	3	0	0	0	0	0	0	0	0
Tochigi	1,942,494	2	4	4	1	2	0	0	1	0
Gunma	1,943,667	6	3	3	0	0	0	0	0	3
Saitama	7,385,848	10	2	2	1	0	0	1	0	0
Chiba	6,310,875	20	7	5	1	1	2	1	0	0
Tokyo	13,794,933	38	14	12	3	2	1	1	0	5
Kanagawa	9,215,210	16	2	2	0	0	0	0	0	2
Niigata	2,188,469	5	1	0	0	0	0	0	0	0
Toyama	1,037,319	5	1	1	1	0	0	0	0	0
Ishikawa	1,124,501	7	0	0	0	0	0	0	0	0
Fukui	767,561	5	0	0	0	0	0	0	0	0
Yamanashi	816,340	2	0	0	0	0	0	0	0	0
Nagano	2,056,970	3	3	3	1	1	1	0	0	0
Gifu	1,996,682	6	1	1	1	0	0	0	0	0
Shizuoka	3,658,375	11	2	2	0	0	0	0	0	2
Aichi	7,528,519	13	2	2	1	1	0	0	0	0
Mie	1,784,968	3	0	0	0	0	0	0	0	0
Shiga	1,415,222	2	0	0	0	0	0	0	0	0
Kyoto	2,511,494	6	1	1	1	0	0	0	0	0
Osaka	8,800,753	21	17	12	2	1	5	1	0	3
Hyogo	5,488,605	16	2	2	0	0	1	1	0	0
Nara	1,335,378	5	2	1	0	1	0	0	0	0
Wakayama	935,084	2	1	1	0	0	1	0	0	0
Tottori	551,806	1	0	0	0	0	0	0	0	0
Shimane	666,331	3	0	0	0	0	0	0	0	0
Okayama	1,879,280	10	0	0	0	0	0	0	0	0
Hiroshima	2,788,687	4	0	0	0	0	0	0	0	0
Yamaguchi	1,340,458	2	0	0	0	0	0	0	0	0
Tokushima	726,729	2	1	1	0	0	0	0	0	1
Kagawa	964,885	4	0	0	0	0	0	0	0	0
Ehime	1,341,539	2	0	0	0	0	0	0	0	0
Kochi	693,369	3	1	0	0	0	0	0	0	0
Fukuoka	5,108,507	19	8	7	1	2	1	1	0	2
Saga	812,193	1	2	2	1	0	0	1	0	0
Nagasaki	1,320,055	7	4	3	1	1	0	0	0	1
Kumamoto	1,747,513	7	5	5	0	0	0	0	0	5
Oita	1,131,140	4	0	0	0	0	0	0	0	0
Miyazaki	1,078,313	4	5	3	0	0	0	0	0	3
Kagoshima	1,605,419	4	1	1	0	0	1	0	0	0
Okinawa	1,485,670	4	0	0	0	0	0	0	0	0

No regional species bias was observed（$p = 0.457$）.

[a] Names of regions in Japan are arranged in order from north to south in this table.

[b] Population data are based on demographic information published by the Ministry of Internal Affairs and Communications of Japan.

Table S2. The number of patients per year by organ, the reasons for the diagnosis, the causative organisms, and the reasons for the identification of the causative organisms.

	Pulmonary n=58		Sinus n=16		Skin n=13		Cerebral n=9		Blood n=8		Gastrointestinal tissue n=7		Other organs n=14 [a]		Disseminated n=29 [b]	
Year, n (%)																
2015	7	(12.1%)	4	(25.0%)	1	(7.7%)	2	(22.2%)	1	(12.5%)	0	(0.0%)	1	(7.1%)	3	(10.3%)
2016	12	(20.7%)	1	(6.3%)	2	(15.4%)	1	(11.1%)	0	(0.0%)	2	(28.6%)	5	(35.7%)	5	(17.2%)
2017	8	(13.8%)	1	(6.3%)	2	(15.4%)	2	(22.2%)	0	(0.0%)	0	(0.0%)	2	(14.3%)	4	(13.8%)
2018	5	(8.6%)	1	(6.3%)	1	(7.7%)	0	(0.0%)	2	(25.0%)	0	(0.0%)	1	(7.1%)	3	(10.3%)
2019	10	(17.2%)	6	(37.5%)	2	(15.4%)	2	(22.2%)	1	(12.5%)	0	(0.0%)	2	(14.3%)	6	(20.7%)
2020	7	(12.1%)	3	(18.8%)	3	(23.1%)	2	(22.2%)	2	(25.0%)	2	(28.6%)	1	(7.1%)	4	(13.8%)
2021	5	(8.6%)	0	(0.0%)	1	(7.7%)	0	(0.0%)	1	(12.5%)	2	(28.6%)	2	(14.3%)	2	(6.9%)
2022	4	(6.9%)	0	(0.0%)	1	(7.7%)	0	(0.0%)	1	(12.5%)	1	(14.3%)	0	(0.0%)	2	(6.9%)
Proven mucormycosis, n (%) [c]	43	(74.1%)	10	(62.5%)	13	(100.0%)	8	(88.9%)	8	(100.0%)	7	(100.0%)	14	(100.0%)	25	(86.2%)
The rationale for “proven”, n (%)																
Positive sterile material microscopic analysis [d]	34	(58.6%)	9	(56.3%)	13	(100.0%)	7	(77.8%)	3	(37.5%)	7	(100.0%)	13	(92.9%)	22	(75.9%)
A positive culture from sterile material [e]	30	(51.7%)	3	(18.8%)	5	(38.5%)	4	(44.4%)	8	(100.0%)	4	(57.1%)	9	(64.3%)	14	(48.3%)
Probable mucormycosis, n (%) [f]	15	(25.9%)	6	(37.5%)	0	(0.0%)	1	(11.1%)	0	(0.0%)	0	(0.0%)	0	(0.0%)	4	(13.8%)
Three elements used to determine “probable”, n (%) [g]																
Patients with host factors	53	(91.4%)	12	(75.0%)	12	(92.3%)	8	(88.9%)	5	(62.5%)	6	(85.7%)	11	(78.6%)	26	(89.7%)
Patients with clinical features	56	(96.6%)	15	(93.8%)	8	(61.5%)	7	(77.8%)	5	(62.5%)	3	(42.9%)	11	(78.6%)	24	(82.8%)
Patients with mycological evidence	58	(100.0%)	16	(100.0%)	13	(100.0%)	9	(100.0%)	8	(100.0%)	7	(100.0%)	14	(100.0%)	29	(100.0%)
Organism, n (%) [h]																
Rhizopus spp.	11	(19.0%)	4	(25.0%)	3	(23.1%)	2	(22.2%)	0	(0.0%)	2	(28.6%)	2	(14.3%)	5	(17.2%)
Cunninghamella spp.	12	(20.7%)	0	(0.0%)	3	(23.1%)	2	(22.2%)	0	(0.0%)	1	(14.3%)	4	(28.6%)	4	(13.8%)
Mucor spp.	7	(12.1%)	5	(31.3%)	2	(15.4%)	0	(0.0%)	5	(62.5%)	1	(14.3%)	0	(0.0%)	5	(17.2%)
Rhizomucor spp.	6	(10.3%)	1	(6.3%)	1	(7.7%)	0	(0.0%)	0	(0.0%)	1	(14.3%)	1	(7.1%)	3	(10.3%)
Lichtheimia spp.	1	(1.7%)	0	(0.0%)	0	(0.0%)	0	(0.0%)	1	(12.5%)	0	(0.0%)	0	(0.0%)	1	(3.4%)
Genus not identified	22	(37.9%)	6	(37.5%)	4	(30.8%)	5	(55.6%)	2	(25.0%)	2	(28.6%)	7	(50.0%)	11	(37.9%)
Organism identification, n (%) [i]																
with culture morphology	24	(41.4%)	7	(43.8%)	7	(53.8%)	2	(22.2%)	5	(62.5%)	3	(42.9%)	3	(21.4%)	11	(37.9%)
with genetic identification	20	(34.5%)	4	(25.0%)	5	(38.5%)	4	(44.4%)	2	(25.0%)	3	(42.9%)	4	(28.6%)	10	(34.5%)
with time-of-flight mass spectrometry	7	(12.1%)	0	(0.0%)	2	(15.4%)	2	(22.2%)	1	(12.5%)	1	(14.3%)	2	(14.3%)	3	(10.3%)

Percentages were calculated based on the number of patients in each group. The number of infection sites and causative organisms includes duplicates. No cases of orbital lesions were identified.

a. Other organs included the thyroid, bronchus, heart, pleura, spine, liver, pancreas, spleen, kidney, bladder, and retroperitoneum.

b. Cases with confirmed infection of two or more organs were defined as disseminated.

c. All patients with proven mucormycosis here meet one or more of the criteria for the rationale for proven.

d. Histopathologic, cytopathologic, or direct microscopic examination of a specimen obtained by needle aspiration or biopsy in which hyphae are seen accompanied by evidence of associated tissue damage.

e. Recovery of a mold by the culture of a specimen obtained by a sterile procedure from a normally sterile and clinically or radiologically abnormal site consistent with an infectious disease process,

f. All patients with probable mucormycosis here are immunocompromised and meet the criteria of at least 1 host factor, a clinical feature, and mycologic evidence.

g. The three elements used to determine probable were the definitions provided by the Revision and Update of the Consensus Definitions of Invasive Fungal Disease From the European Organization for Research and Treatment of Cancer and the Mycoses Study Group Education and Research Consortium in the 2020 publication.

h. The test results for the difference in the distribution of *Mucorale* genera per infected organ were as follows: Pulmonary ($p = 0.227$), Sinus ($p = 0.324$), Skin ($p = 0.983$), Cerebral ($p = 0.499$), Blood ($p < 0.001$), Gastrointestinal ($p = 0.978$), and Disseminated ($p = 0.737$). The distribution of *Mucorale* genera showed a significant difference only in blood.

i. If more than one identification method was used, each was counted.

Table S3. Comparison of characteristics of mucormycosis patients by organ.

	Pulmonary n=58		Sinus n=16		Skin n=13		Cerebral n=9		Blood n=8		Gastrointestinal tissue n=7		Other organs n=14 [a]		Disseminated n=29 [b]	
Age, n (%)																
0	0	(0.0%)	2	(12.5%)	2	(15.4%)	0	(0.0%)	1	(12.5%)	1	(14.3%)	0	(0.0%)	2	(6.9%)
1-12	3	(5.2%)	2	(12.5%)	1	(7.7%)	0	(0.0%)	2	(25.0%)	0	(0.0%)	0	(0.0%)	2	(6.9%)
13-17	3	(5.2%)	0	(0.0%)	3	(23.1%)	1	(11.1%)	0	(0.0%)	3	(42.9%)	2	(14.3%)	3	(10.3%)
18-64	29	(50.0%)	7	(43.8%)	2	(15.4%)	5	(55.6%)	1	(12.5%)	2	(28.6%)	3	(21.4%)	11	(37.9%)
≥65	23	(39.7%)	5	(31.3%)	5	(38.5%)	3	(33.3%)	4	(50.0%)	1	(14.3%)	9	(64.3%)	11	(37.9%)
Male, n (%)	40	(69.0%)	8	(50.0%)	8	(61.5%)	6	(66.7%)	5	(62.5%)	6	(85.7%)	10	(71.4%)	18	(62.1%)
Underlying condition at the time of diagnosis, n (%) [c]																
Neutrophil <500 /µL for ≥10 days	17	(29.3%)	5	(31.3%)	5	(38.5%)	3	(33.3%)	2	(25.0%)	3	(42.9%)	4	(28.6%)	11	(37.9%)
Neutrophil <500 /µL for ≥30 days	13	(22.4%)	2	(12.5%)	2	(15.4%)	2	(22.2%)	1	(12.5%)	2	(28.6%)	4	(28.6%)	6	(20.7%)
Hematologic malignancy	46	(79.3%)	12	(75.0%)	9	(69.2%)	7	(77.8%)	4	(50.0%)	2	(28.6%)	8	(57.1%)	21	(72.4%)
Allogeneic stem cell transplant	20	(34.5%)	6	(37.5%)	3	(23.1%)	1	(11.1%)	2	(25.0%)	2	(28.6%)	3	(21.4%)	9	(31.0%)
Acute graft-versus-host disease grade I	4	(6.9%)	1	(6.3%)	0	(0.0%)	0	(0.0%)	1	(12.5%)	0	(0.0%)	0	(0.0%)	2	(6.9%)
Acute graft-versus-host disease grade II	5	(8.6%)	1	(6.3%)	0	(0.0%)	0	(0.0%)	0	(0.0%)	0	(0.0%)	1	(7.1%)	1	(3.4%)
Acute graft-versus-host disease grade III	0	(0.0%)	0	(0.0%)	0	(0.0%)	0	(0.0%)	0	(0.0%)	0	(0.0%)	0	(0.0%)	0	(0.0%)
Acute graft-versus-host disease grade IV	2	(3.4%)	0	(0.0%)	1	(7.7%)	0	(0.0%)	0	(0.0%)	0	(0.0%)	0	(0.0%)	0	(0.0%)
Solid organ transplant	3	(5.2%)	0	(0.0%)	0	(0.0%)	1	(11.1%)	0	(0.0%)	1	(14.3%)	1	(7.1%)	1	(3.4%)
Prolonged use of corticosteroids [d]	27	(46.6%)	8	(50.0%)	5	(38.5%)	4	(44.4%)	2	(25.0%)	3	(42.9%)	6	(42.9%)	13	(44.8%)
T-cell immunosuppressants [e]	22	(37.9%)	4	(25.0%)	4	(30.8%)	1	(11.1%)	0	(0.0%)	3	(42.9%)	5	(35.7%)	10	(34.5%)
B-cell immunosuppressants [f]	4	(6.9%)	2	(12.5%)	1	(7.7%)	0	(0.0%)	0	(0.0%)	0	(0.0%)	0	(0.0%)	2	(6.9%)
Persistent hyperglycemia	9	(15.5%)	4	(25.0%)	2	(15.4%)	4	(44.4%)	2	(25.0%)	1	(14.3%)	3	(21.4%)	7	(24.1%)
Metabolic acidosis	6	(10.3%)	1	(6.3%)	1	(7.7%)	1	(11.1%)	1	(12.5%)	0	(0.0%)	2	(14.3%)	5	(17.2%)
Undergoing treatment in the intensive care unit	17	(29.3%)	2	(12.5%)	8	(61.5%)	2	(22.2%)	2	(25.0%)	5	(71.4%)	7	(50.0%)	12	(41.4%)
Undergoing treatment of the iron overload	6	(10.3%)	2	(12.5%)	2	(15.4%)	1	(11.1%)	0	(0.0%)	0	(0.0%)	2	(14.3%)	4	(13.8%)
Undergoing treatment for the COVID-19	1	(1.7%)	0	(0.0%)	0	(0.0%)	0	(0.0%)	1	(12.5%)	1	(14.3%)	0	(0.0%)	1	(3.4%)
Neutrophil recovered to >1000/µL, n (%) [g]																
Already ≥1000/µL at diagnosis	36	(62.1%)	9	(56.3%)	8	(61.5%)	5	(55.6%)	5	(62.5%)	4	(57.1%)	8	(57.1%)	13	(44.8%)
Recovered within 7 days of diagnosis	2	(3.4%)	1	(6.3%)	0	(0.0%)	0	(0.0%)	1	(12.5%)	0	(0.0%)	0	(0.0%)	2	(6.9%)
Recovered within 8-14 days of diagnosis	2	(3.4%)	0	(0.0%)	4	(30.8%)	1	(11.1%)	1	(12.5%)	1	(14.3%)	0	(0.0%)	4	(13.8%)
Recovered after 15 days from diagnosis	0	(0.0%)	1	(6.3%)	0	(0.0%)	0	(0.0%)	0	(0.0%)	1	(14.3%)	0	(0.0%)	0	(0.0%)
No recovery to >1000 neutrophils by the end of observation	16	(27.6%)	5	(31.3%)	1	(7.7%)	3	(33.3%)	0	(0.0%)	1	(14.3%)	6	(42.9%)	9	(31.0%)

Percentages were calculated based on the number of patients in each group. Cases for each organ include duplicates. There were no cases of orbital lesions. The definition of 'time of diagnosis' here refers to the date on which the specimen with *Mucorales* was collected.

a. Other organs included the thyroid, bronchus, heart, pleura, spine, liver, pancreas, spleen, kidney, bladder, and retroperitoneum.

b. Cases with confirmed infection of two or more organs were defined as disseminated.

c. There were no patients with inherited severe immunodeficiency or human immunodeficiency virus infection.

d. Prolonged use of corticosteroids at a therapeutic dose of ≥0.3 mg/kg corticosteroids for ≥3 weeks in the past 60 days.

e. T-cell immunosuppressants, such as calcineurin inhibitors, tumor necrosis factor-a blockers, lymphocyte-specific monoclonal antibodies, and immunosuppressive nucleoside analogs during the past 90 days.

f. B-cell immunosuppressants, such as Bruton’s tyrosine kinase inhibitors, e.g., ibrutinib.

g. Neutrophil status data were not available in 2 cases.

Table S4. Comparison of mucormycosis treatment characteristics by organ.

	Pulmonary n=58	Sinus n=16	Skin n=13	Cerebral n=9	Blood n=8	Gastrointestinal tissue n=7	Other organs n=14 [a]	Disseminated n=29 [b]
Resection of infected lesions, n (%)								
within 3 days of diagnosis	14 (24.1%)	4 (25.0%)	2 (15.4%)	1 (11.1%)	0 (0.0%)	2 (28.6%)	1 (7.1%)	1 (3.4%)
within 4-7 days of diagnosis	1 (1.7%)	0 (0.0%)	1 (7.7%)	0 (0.0%)	0 (0.0%)	0 (0.0%)	0 (0.0%)	0 (0.0%)
within 8-14 days of diagnosis	1 (1.7%)	2 (12.5%)	0 (0.0%)	0 (0.0%)	0 (0.0%)	0 (0.0%)	0 (0.0%)	1 (3.4%)
after 15 days of diagnosis	2 (3.4%)	1 (6.3%)	1 (7.7%)	0 (0.0%)	1 (12.5%)	0 (0.0%)	0 (0.0%)	1 (3.4%)
No resection	40 (69.0%)	9 (56.3%)	9 (69.2%)	8 (88.9%)	7 (87.5%)	5 (71.4%)	13 (92.9%)	26 (89.7%)
Antifungal drugs already used at diagnosis, n (%) [c]								
Fluconazole	3 (5.2%)	1 (6.3%)	2 (15.4%)	2 (22.2%)	1 (12.5%)	1 (14.3%)	2 (14.3%)	4 (13.8%)
Itraconazole	5 (8.6%)	2 (12.5%)	0 (0.0%)	1 (11.1%)	1 (12.5%)	0 (0.0%)	1 (7.1%)	3 (10.3%)
Voriconazole	15 (25.9%)	4 (25.0%)	2 (15.4%)	3 (33.3%)	0 (0.0%)	1 (14.3%)	2 (14.3%)	6 (20.7%)
Posaconazole	2 (3.4%)	0 (0.0%)	0 (0.0%)	0 (0.0%)	0 (0.0%)	0 (0.0%)	1 (7.1%)	0 (0.0%)
Micafungin	7 (12.1%)	3 (18.8%)	4 (30.8%)	1 (11.1%)	2 (25.0%)	2 (28.6%)	1 (7.1%)	6 (20.7%)
Caspofungin	8 (13.8%)	0 (0.0%)	3 (23.1%)	0 (0.0%)	0 (0.0%)	1 (14.3%)	3 (21.4%)	3 (10.3%)
Liposomal amphotericin B	20 (34.5%)	2 (12.5%)	4 (30.8%)	4 (44.4%)	0 (0.0%)	1 (14.3%)	5 (35.7%)	8 (27.6%)
Antifungal drugs administered as a treatment for mucormycosis, n (%) [c]								
Liposomal amphotericin B alone	35 (60.3%)	8 (50.0%)	8 (61.5%)	5 (55.6%)	7 (87.5%)	6 (85.7%)	7 (50.0%)	19 (65.5%)
Posaconazole [d]	3 (5.2%)	0 (0.0%)	0 (0.0%)	0 (0.0%)	0 (0.0%)	0 (0.0%)	0 (0.0%)	0 (0.0%)
Liposomal amphotericin B + posaconazole	1 (1.7%)	1 (6.3%)	0 (0.0%)	0 (0.0%)	0 (0.0%)	0 (0.0%)	1 (7.1%)	1 (3.4%)
Liposomal amphotericin B + micafungin	2 (3.4%)	1 (6.3%)	0 (0.0%)	0 (0.0%)	0 (0.0%)	0 (0.0%)	0 (0.0%)	1 (3.4%)
Liposomal amphotericin B + caspofungin	5 (8.6%)	1 (6.3%)	1 (7.7%)	2 (22.2%)	0 (0.0%)	0 (0.0%)	1 (7.1%)	2 (6.9%)
Liposomal amphotericin B + others [e]	3 (5.2%)	3 (18.8%)	2 (15.4%)	1 (11.1%)	0 (0.0%)	0 (0.0%)	1 (7.1%)	2 (6.9%)
No effective antifungal administration [f]	9 (15.5%)	2 (12.5%)	2 (15.4%)	1 (11.1%)	1 (12.5%)	1 (14.3%)	4 (28.6%)	4 (13.8%)
Maximum dose of liposomal amphotericin B, n (%)								
No administration	12 (20.7%)	2 (12.5%)	2 (15.4%)	1 (11.1%)	1 (12.5%)	1 (14.3%)	4 (28.6%)	4 (13.8%)
<5mg/kg	8 (13.8%)	4 (25.0%)	1 (7.7%)	2 (22.2%)	1 (12.5%)	0 (0.0%)	2 (14.3%)	5 (17.2%)
5mg/kg	28 (48.3%)	6 (37.5%)	5 (38.5%)	1 (11.1%)	3 (37.5%)	6 (85.7%)	6 (42.9%)	12 (41.4%)
6mg/kg	3 (5.2%)	2 (12.5%)	1 (7.7%)	2 (22.2%)	1 (12.5%)	0 (0.0%)	0 (0.0%)	3 (10.3%)
7mg/kg	1 (1.7%)	0 (0.0%)	1 (7.7%)	1 (11.1%)	0 (0.0%)	0 (0.0%)	0 (0.0%)	1 (3.4%)
8mg/kg	2 (3.4%)	0 (0.0%)	2 (15.4%)	1 (11.1%)	0 (0.0%)	0 (0.0%)	1 (7.1%)	1 (3.4%)
9mg/kg	1 (1.7%)	1 (6.3%)	0 (0.0%)	0 (0.0%)	0 (0.0%)	0 (0.0%)	1 (7.1%)	1 (3.4%)
≥10mg/k	3 (5.2%)	1 (6.3%)	1 (7.7%)	1 (11.1%)	2 (25.0%)	0 (0.0%)	0 (0.0%)	2 (6.9%)
Start liposomal amphotericin B administration at ≥5 mg/kg, n (%)								
before diagnosis	18 (31.0%)	1 (6.3%)	2 (15.4%)	1 (11.1%)	0 (0.0%)	1 (14.3%)	3 (21.4%)	4 (13.8%)
within 3 days of diagnosis	11 (19.0%)	7 (43.8%)	6 (46.2%)	3 (33.3%)	4 (50.0%)	2 (28.6%)	1 (7.1%)	10 (34.5%)
within 4-7 days of diagnosis	8 (13.8%)	0 (0.0%)	0 (0.0%)	1 (11.1%)	1 (12.5%)	1 (14.3%)	3 (21.4%)	4 (13.8%)
within 8-14 days of diagnosis	1 (1.7%)	3 (18.8%)	2 (15.4%)	0 (0.0%)	1 (12.5%)	2 (28.6%)	1 (7.1%)	3 (10.3%)
after 15 days of diagnosis	2 (3.4%)	1 (6.3%)	0 (0.0%)	1 (11.1%)	1 (12.5%)	0 (0.0%)	0 (0.0%)	1 (3.4%)
No administration of liposomal amphotericin B at ≥5 mg/kg	18 (31.0%)	4 (25.0%)	3 (23.1%)	3 (33.3%)	1 (12.5%)	1 (14.3%)	6 (42.9%)	7 (24.1%)

Percentages were calculated based on the number of patients in each group. Cases for each organ include duplicates. No cases of orbital lesions were identified. The definition of "diagnosis" here refers to the date on which the specimen with *Mucorales* was collected.

a. Other organs included the thyroid, bronchus, heart, pleura, spine, liver, pancreas, spleen, kidney, bladder, and retroperitoneum.

b. Cases with confirmed infection of two or more organs were defined as disseminated.

c. Isavuconazole was not approved in Japan at the time of data collection.

d. One case of a combination of posaconazole and caspofungin was included.

e. Other antifungals used in combination were fluconazole and itraconazole in 1 case each and voriconazole in 4 cases.

f. Patients not receiving liposomal amphotericin B or posaconazole.

Table S5. Prognosis of Mucormycosis Patients

	Pulmonary n=58		Sinus n=16		Skin n=13		Cerebral n=9		Blood n=8		Gastrointestinal tissue n=7		Other organs n=14 [a]		Disseminated n=29 [b]	
Treatment discontinuation due to liposomal amphotericin B intolerance, n (%)	6	(10.3%)	0	(0.0%)	0	(0.0%)	1	(11.1%)	0	(0.0%)	2	(28.6%)	3	(21.4%)	2	(6.9%)
Non-survivors, n (%)																
as diagnosed at post-mortem examination	6	(10.3%)	1	(6.3%)	1	(7.7%)	2	(22.2%)	0	(0.0%)	2	(28.6%)	5	(35.7%)	5	(17.2%)
within 4 weeks	22	(37.9%)	6	(37.5%)	6	(46.2%)	5	(55.6%)	2	(25.0%)	5	(71.4%)	8	(57.1%)	15	(51.7%)
within 6 weeks	25	(43.1%)	6	(37.5%)	6	(46.2%)	5	(55.6%)	2	(25.0%)	5	(71.4%)	9	(64.3%)	16	(55.2%)
within 12 weeks	28	(48.3%)	7	(43.8%)	7	(53.8%)	5	(55.6%)	3	(37.5%)	6	(85.7%)	11	(78.6%)	17	(58.6%)

Cases for each organ include duplicates. There were no cases of orbital lesions. Percentages were calculated based on the number of patients in each group.

a. Other organs included the thyroid, bronchus, heart, pleura, spine, liver, pancreas, spleen, kidney, bladder, and retroperitoneum.

b. Cases with confirmed infection of two or more organs were defined as disseminated.

c. In the "other organs" category, cases who were diagnosed at post-mortem examination ($p = 0.006$) and non-survivors within 12 weeks of diagnosis ($p = 0.0409$) were significantly more common. However, no significant differences were found in the other combinations.

Table S6. Cox regression analysis of factors associated with 4 weeks mortality in mucormycosis patients treated with liposomal amphotericin B

Factor	Hazard Ratio	95% Confidence Interval	*P* value
Treated with >5 mg/kg of liposomal amphotericin B [a]	0.86	(0.28 –2.68)	0.796
Older adults (65 years and older)	0.58	(0.12 –2.82)	0.504
No recovery to >1000 neutrophils by the end of observation	5.82	(1.84 –18.44)	0.003
Resection of infected lesions	0.11	(0.01 –0.88)	0.038

Data apply to 49 patients who were subjects for analysis of the efficacy of liposomal amphotericin B treatment.

[a] The reference category was set at patients treated with 5 mg/kg liposomal amphotericin B.

Table S7. Research Collaboration Institutions

Prefectural Regions in Japan	Name of Institution	Name of Department
Hokkaido	Asahikawa Medical University Hospital	Department of Pediatrics
Hokkaido	Hokkaido University Hospital	Division of Infection Control
Hokkaido	Japanese Red Cross Kitami Hospital	Department of Internal Medicine
Hokkaido	Japanese Red Cross Society Red Cross Hospital HAKODATE	Department of Hematology and Oncology
Hokkaido	Kushiro Rosai Hospital	Department of Hematology
Hokkaido	Nayoro City General Hospital	Department of Pediatrics
Hokkaido	Obihiro Kosei Hospital	Department of Respiratory Medicine
Hokkaido	Sapporo Hokuyu Hospital	Department of Pediatrics
Hokkaido	Sapporo Kiyota Hospital	Department of Hematology
Hokkaido	Sapporo Kosei General Hospital	Department of Hematology
Hokkaido	Sapporo Medical University Hospital	Department of Pediatrics
Hokkaido	Sapporo Medical University Hospital	Division of Infection Control
Hokkaido	Sapporo Tokushukai Hospital	Department of Pediatrics
Hokkaido	Sapporo Medical University Hospital	Department of Respiratory Medicine
Hokkaido	Teine Keijinkai Hospital	Department of Hematology
Iwate	Iwate Medical University Hospital	Department of Pediatrics
Iwate	Iwate Medical University Hospital	Division of Hematology and Oncology
Iwate	Iwate Prefectural Isawa Hospital	Department of Hematology
Miyagi	Miyagi Children's Hospital	Department of Hematology & Oncology
Miyagi	National Hospital Organization Sendai Medical Center	Department of Hematology
Miyagi	Sendai City Hospital	Department of Hematology
Miyagi	Sendai City Hospital	Division of Infectious Diseases
Miyagi	Tohoku University Hospital	Department of Clinical Infectious Diseases
Miyagi	Tohoku University Hospital	Department of Respiratory Medicine
Akita	Akita City Hospital	Department of Hematology and Nephrology
Akita	Kotou Kousei Hospital	Department of Internal Medicine
Akita	Noshiro Kosei Medical Center	Department of Hematology
Yamagata	Miyukikai Hospital	Department of Internal Medicine
Yamagata	Okitama Public General Hospital	Department of Hematology
Yamagata	Yamagata Prefectural Central Hospital	Department of Hematology
Yamagata	Yamagata University Hospital	Department of Pediatrics
Fukushima	Aizu Medical Center	Department of Hematology
Fukushima	Fukushima Medical University Hospital	Department of Hematology
Fukushima	Fukushima Medical University Hospital	Department of Pediatric Oncology
Fukushima	Minamisoma City General Hospital	Department of Hematology
Ibaraki	Tsukuba Medical Center Hospital	Division of Infectious Diseases, Department of Medicine
Ibaraki	Tsukuba Memorial Hospital	Department of Hematology
Ibaraki	University of Tsukuba Hospital	Department of Hematology
Tochigi	Dokkyo Medical University Hospital	Department of Hematology and Oncology
Tochigi	Dokkyo Medical University Hospital	Department of Pediatrics
Tochigi	Jichi Medical University Hospital	Department of Dermatology
Tochigi	Jichi Medical University Hospital	Division of Hematology
Gunma	Fujioka General Hospital	Department of Hematology
Gunma	Gunma Childrens Medical Center	Department of Infectious Diseases
Gunma	Gunma University Hospital	Department of Hematology
Gunma	Gunma University Hospital	Department of Ophthalmology
Gunma	Gunma University Hospital	Department of Pediatrics
Gunma	Gunma University Hospital	Infection Control and Prevention Center
Gunma	Hoshi clinic	Department of Hematology
Gunma	Maebashi Red Cross Hospital	Department of Internal Medicine of Hematology
Gunma	Saiseikai Maebashi Hospital	Department of Hematology
Saitama	Ageo Central General Hospital	Department of General Medicine
Saitama	Jichi Medical University Saitama Medical Center	Department of Dermatology
Saitama	Jichi Medical University Saitama Medical Center	Division of General Medicine
Saitama	Jichi Medical University Saitama Medical Center	Division of Hematology
Saitama	Saiseikai General Hospital	Department of Dermatology
Saitama	Saitama Cancer Center	Division of Hematology
Saitama	Saitama Citizens Medical Center	Department of Hematology
Saitama	Saitama City Hospital	Division of Infectious Diseases
Saitama	Saitama Medical University Hospital	Department of Dermatology
Saitama	Saitama Medical University International Medical Center	Department of Hematooncology
Saitama	Saitama Medical University International Medical Center	Department of Infectious Diseases and Infection Control
Saitama	Saitama Medical University International Medical Center	Department of Pediatric Hematology and Oncology
Saitama	Saitama Medical University Saitama Medical Center	Department of Dermatology
Saitama	TMG Asaka Medical Center	Department of Hematology
Chiba	Asahi General Hospital	Department of Hematology
Chiba	Chiaken Saiseikai Narashino Hospital	Department of Respiratory Medicine
Chiba	Chiba Aoba Municipal Hospital	Department of Respiratory Medicine
Chiba	Chiba Cancer Center	Department of Hematology and Medical Oncology
Chiba	Chiba Children's Hospital	Department of Hematology and Oncology

Chiba	Chiba University Hospital	Department of Hematology
Chiba	Inoue Memorial Hospital	Department of Internal Medicine
Chiba	Japanese Red Cross Narita Hospital	Department of Pediatrics
Chiba	Kameda Medical Center	Division of Infectious Diseases
Chiba	Kashiwa Kousei General Hospital	Department of Hematology and Oncology
Chiba	Kohnodai Hospital, National Center for Global Health and Medicine	Department of Internal Medicine
Chiba	Narita Memorial Hospital	Department of Pediatrics
Chiba	National Hospital Organization Chiba Medical Center	Department of Internal Medicine
Chiba	National Insurance General Hospital Kimitsu Central Hospital	Department of Dermatology
Chiba	Nippon Medical School Chiba Hokusoh Hospital	Department of Dermatology
Chiba	Nippon Medical School Chiba Hokusoh Hospital	Department of Hematology
Chiba	Teikyo University Chiba Medical Center	Department of Dermatology
Chiba	The Jikei University Kashiwa Hospital	Department of Infectious Diseases and Infection Control
Chiba	The Jikei University Kashiwa Hospital	Department of Respiratory Medicine
Chiba	Toho University Medical Center Sakura Hospital	Department of Hematology
Chiba	Tokyo Bay Urayasu Ichikawa Medical Center	Department of Infectious Diseases
Chiba	Tokyo Dental College Ichikawa General Hospital	Department of respiratory medicine
Tokyo	Eiju General Hospital	Department of Hematology
Tokyo	IUHW Mita Hospital	Department of Hematology
Tokyo	Japanese Red Cross Medical Center	Department of Infectious Diseases
Tokyo	JCHO Tokyo Yamate Medical Center	Department of Respiratory Medicine, Infectious Diseases
Tokyo	Juntendo Tokyo Koto Geriatric Medical Center	Department of Dermatology
Tokyo	Juntendo University Nerima Hospital	Department of Dermatology and Allergology
Tokyo	Juntendo University Nerima Hospital	Department of Hematology
Tokyo	Keio University, School of Medicine	Department of Infectious Diseases
Tokyo	Kudanzaka Hospital	Department of Dermatology
Tokyo	Makita General Hospital	Department of Dermatology
Tokyo	Mishuku Hospital	Department of Hematology
Tokyo	Moriyama Memorial Hospital	Department of Internal Medicine
Tokyo	National Cancer Center Hospital	Department of Pediatric Oncology
Tokyo	National Center for Global Health and Medicine	AIDS Clinical Center
Tokyo	National Center for Global Health and Medicine	Department of Pediatrics
Tokyo	National Hospital Organization Tokyo Medical Center	Department of General Medicine
Tokyo	National Hospital Organization Tokyo National Hospital	Center for Pulmonary Diseases
Tokyo	Nerima General Hospital	Department of Pharmacy
Tokyo	Nihon University Hospital	Division of Hematology and Rheumatology
Tokyo	Nippon Medical School Hospital	Department of Hematology
Tokyo	Nippon Medical School Hospital	Department of Pediatrics
Tokyo	Showa General Hospital	Department of Pediatrics
Tokyo	Showa University Hospital	Department of Pediatrics
Tokyo	Showa University Hospital	Division of Clinical Infectious Diseases, Department of Medicine, School of Medicine
Tokyo	Showa University Hospital	Division of Hematology, Department of Medicine
Tokyo	Showa University Koto Toyosu Hospital	Department of Pediatrics
Tokyo	St. Lule's International Hospital	Department of Infectious Diseases
Tokyo	St. Lule's International Hospital	Department of Pediatrics
Tokyo	Teikyo University Hospital	Department of Dermatology
Tokyo	Teikyo University Hospital	Department of Hematology and Oncology
Tokyo	Teikyo University Hospital	Department of Pediatrics
Tokyo	The Jikei University Hospital	Department of Hematology and Oncology
Tokyo	The Jikei University Hospital	Department of Infectious Diseases and Infection Control
Tokyo	The Jikei University Hospital	Department of Pediatrics
Tokyo	The Jikei University Hospital	Department of Respiratory Medicine
Tokyo	The Jikei University Katsushika Medical Center	Department of Infectious Disease and Infection Control
Tokyo	Toho University Ohashi Medical Center	Department of Otorhinolaryngology
Tokyo	Toho University Omori Medical Center	Department of Otorhinolaryngology
Tokyo	Toho University Omori Medical Center	Department of Pediatrics
Tokyo	Tokyo Medical and Dental University Hospital	Department of Pediatrics
Tokyo	Tokyo Medical University Hachioji Medical Center	Department of Clinical Infectious Diseases
Tokyo	Tokyo Metropolitan Bokutoh Hospital	Department of Infectious Diseases and Department of Hematology
Tokyo	Tokyo Metropolitan Geriatric Hospital	Division of Infectious Diseases
Tokyo	Tokyo Shinagawa Hospital	Department of Hematology
Tokyo	Tokyo Teishin Hospital	Department of Hematology
Tokyo	Tokyo Teishin Hospital	Division of Infectious Diseases
Tokyo	Tokyo Women's Medical University Hospital	Department of Pediatrics
Tokyo	Tokyo-kyosai Hospital	Division of Hematology
Tokyo	University of Tokyo Hospital	Department of Infectious Diseases
Tokyo	University of Tokyo Hospital	Department of Pediatrics
Tokyo	Yurin Hospital	Department of Internal Medicine
Kanagawa	Hiratsuka City Hospital	Department of Dermatology
Kanagawa	Kanagawa Cardiovascular and Respiratory Center	Department of Respiratory Medicine
Kanagawa	Kanagawa Children's Medical Center	Department of Hematology and Oncology
Kanagawa	Kawasaki Municipal Tama Hospital	Department of Neurosurgery
Kanagawa	Kawasaki Municipal Hospital	Department of Infectious Diseases

Kanagawa	Nippon Koukan Hospital	Department of Internal Medicine
Kanagawa	Nippon Medical School Musashikosugi Hospital	Department of Dermatology
Kanagawa	Saiseikai Yokohama Nanbu Hospital	Department of Pediatrics
Kanagawa	Shonan-Fujisawa Tokushukai Hospital	Department of Dermatology
Kanagawa	Showa University Northern Yokohama Hospital	Department of Internal Medicine
Kanagawa	St. Marianna University School of Medicine Hospital	Department of Hematology and Oncology
Kanagawa	St. Marianna University School of Medicine Hospital	Department of Respiratory Medicine
Kanagawa	St. Marianna University Yokohama Seibu Hospital	Department of Hematology
Kanagawa	Teikyo University School of Medicine University Hospital, Mizonokuchi	Department of 4th Internal Medicine
Kanagawa	Tokai University Hospital	Department of Pediatrics
Kanagawa	Yokohama City Minato Red Cross Hospital	Department of Hematology
Kanagawa	Yokohama City Minato Red Cross Hospital	Department of Infectious Diseases
Kanagawa	Yokohama Ekisaikai Hospital	Department of Internal Medicine
Niigata	Nagaoka Red Cross Hospital	Department of Infectious Diseases
Niigata	Niigata City General Hospital	Division of Infectious Diseases
Niigata	Niigata University Medical and Dental Hospital	Department of Pediatrics
Niigata	Niigata University Medical and Dental Hospital	Department of Respiratory Medicine and Infectious Diseases
Niigata	Niigatakenritu tsubame rosai hospital	Department of Internal Medicine
Niigata	Uonuma Kikan Hospital	Department of Hematology
Toyama	JA Toyama Kouseiren Takaoka Hospital	Department of Hematology
Toyama	Shinseikai Toyama Hospital	Department of Dermatology
Toyama	Toyama City Hospital	Division of Hematology
Toyama	Toyama Nishi General Hospital	Department of Internal Medicine
Toyama	Toyama University Hospital	Department of Clinical Infectious Diseases
Ishikawa	Ishikawa Prefectural Central Hospital	Department of Immunology-Infectious Diseases
Ishikawa	Japan Community Health Care Organization Kanazawa Hospital	Department of Respiratory Medicine
Ishikawa	Kanazawa Medical University Hospital	Department of Infectious Diseases
Ishikawa	Keiju General Hospital	Department of Hematology
Ishikawa	Keiju Kanazawa Hospital	Department of Internal Medicine
Ishikawa	Komatsu Municipal Hospital	Department of Internal Medicine
Ishikawa	NHO Kanazawa medical center	Department of Gastroenterology
Fukui	Fukui Prefectural Hospital	Department of Hematology and Oncology
Fukui	National Hospital Organization Awara Hospital	Department of Internal Medicine
Fukui	National Hospital Organization Tsuruga Medical Center	Department of Internal Medicine
Fukui	National Hospital Organization Tsuruga Medical Center	Department of Pediatrics
Fukui	University of Fukui Hospital	Department of Hematology and Oncology
Yamanashi	Japanese Red Cross Society Suwa Hospital	Department of Respiratory Medicine
Yamanashi	University of Yamanashi Hospital	Department of Hematology and Oncology
Nagano	Ina central hospital	Department of Dermatology
Nagano	Nouth Alps Medical Center Azumi Hospital	Department of Hematology
Nagano	Shinshu University Hospital	Department of Pediatrics
Nagano	Shinshu University Hospital	Division of Infection Control
Gifu	Asahi University Hospital	Department of Dermatology
Gifu	Gifu Municipal Hospital	Department of Pediatrics
Gifu	Gifu University Hospital	Center for Nutrition Support & Infection Control
Gifu	Gifu University Hospital	Department of Pediatrics
Gifu	Japanese Red Cross Gifu Hospital	Department of Dermatology
Gifu	National Hospital Organization NAGARA Medical Center	Department of Respiratory Medicine
Gifu	Ogaki Municipal Hospital	Department of Pediatrics
Shizuoka	Hamamatsu Medical Center	Department of Infectious Diseases
Shizuoka	Hamamatsu University Hospital	Department of Pediatrics
Shizuoka	Juntendo University Shizuoka Hospital	Department of Dermatology and Allergology
Shizuoka	National Hospital Organization Shizuoka Medical Center	Department of Dermatology
Shizuoka	Seirei Hamamatsu General Hospital	Department of Hematology
Shizuoka	Seirei Hamamatsu General Hospital	Department of Pediatrics
Shizuoka	Shimada General Medical Center	Department of Hematology
Shizuoka	Shizuoka Cancer Center	Department of Infectious Diseases
Shizuoka	Shizuoka Children's Hospital	Department of Hematology and Oncology
Shizuoka	Shizuoka City Shimizu Hospital	Department of Respiratory Medicine
Shizuoka	Shizuoka General Hospital	Department of Emergency Medicine
Shizuoka	Shizuoka Red Cross Hospital	Department of Hematology
Aichi	Aichi Cancer Center	Division of Infectious Diseases
Aichi	Aichi Medical Hospital	Clinical Department of infectious disease
Aichi	Anjo Kosei Hospital	Department of Pediatrics
Aichi	Fujita Health University Okazaki Medical Center	Department of Hematology and Oncology
Aichi	Nagoya City University Hospital	Department of Infection Prevention & Control
Aichi	Nagoya Memorial Hospital	Department of Pediatrics
Aichi	Nagoya Tokushukai General Hospital	Department of Hematology
Aichi	Nagoya Tokushukai General Hospital	Department of Respiratory Medicine
Aichi	Nagoya University Hospital	Department of Infectious Diseases
Aichi	Nagoya University Hospital	Department of Pediatrics
Aichi	National Hospital Organization Higashi Nagoya National Hospital	Department of Respiratory Medicine
Aichi	National Hospital Organization Nagoya Medical Center	Department of Pediatrics

Aichi	Toyota Kosei Hospital	Department of Hematology
Aichi	Toyota Kosei Hospital	Department of Infectious Diseases
Aichi	Toyota Memorial Hospital	Department of Respiratory Medicine
Aichi	Tsushima Municipal Hospital	Department of Respiratory Medicine
Mie	Ise Municipal General Hospital	Department of Internal Medicine
Mie	Isekeiyu Hospital	Department of Pediatrics
Mie	Takeuchi Hospital	Department of Internal Medicine
Shiga	Otsu Red Cross Hospital	Department of Pediatrics
Shiga	Shiga University of Medical Science Hospital	Department of Pediatrics
Shiga	Shiga University of Medical Science Hospital	Division of Infectious Control and Prevention
Kyoto	Japanese Red Cross Kyoto Daiichi Hospital	Department of Hematology
Kyoto	Kyoto City Hospital	Department of Pediatrics
Kyoto	Kyoto Prefectural University of Medicine	Department of Hematology and Oncology
Kyoto	Kyoto University Hospital	Department of Pediatrics
Kyoto	Kyoto-Katsura Hospital	Department of Hematology
Kyoto	Rakuwakai Otowa Hospital	Department of Infectious Diseases
Osaka	Educational Foundation of Osaka Medical and Pharmaceutical University	The Third Department of Internal Medicine
Osaka	Higashiosaka City Medical Center	Department of Pediatrics
Osaka	Imai City Hospital	Department of Hematology
Osaka	Kansai Medical University Hospital	Department of Hematology
Osaka	Kansai Medical University Hospital	Department of Pediatrics
Osaka	Kindai University Hospital	Department of Medical Safety Management, Division of Infection Control and Prevention
Osaka	NHO Kinki-Chuo Medical Center	Clinical Research Center
Osaka	Osaka Medical and Pharmaceutical University Hospital	Infection Control Center
Osaka	Osaka City General Hospital	Department of Infectious Diseases
Osaka	Osaka City General Hospital	Department of Pediatric Hematology and Oncology
Osaka	Osaka Gyoumeikan Hospital	Department of Pediatrics
Osaka	Osaka Habikino Medical Center	Department of Infectious Diseases
Osaka	Osaka Metropolitan University Hospital	Infection Control Science
Osaka	Osaka Red Cross Hospital	Department of Respiratory Medicine
Osaka	Osaka University Hospital	Department of Infection Control
Osaka	Rinku General Medical Center	Division of General Internal Medicine and Infectious Diseases
Osaka	Saiseikai Ibaraki Hospital	Department of Hematology
Osaka	Saiseikai Nakatsu Hospital	Department of Nephrology
Osaka	Sakai City Medical Center	Department of Infectious Diseases
Osaka	Suita Municipal Hospital	Department of Hematology
Osaka	Teramoto Memorial Hospital	Department of Hematology
Osaka	Toyonaka Municipal Hospital	Department of Hematology
Osaka	Wakakusa Daiichi-Hospital	Department of Internal Medicine
Hyogo	Akashi City Hospital	Business Administration Headquarters
Hyogo	Amagasaki Central Hospital	Department of Internal Medicine
Hyogo	Hyogo Cancer Center	Department of Hematology
Hyogo	Hyogo medical university	Department of Pediatrics
Hyogo	Hyogo Prefectural Amagasaki General Medical Center	Department of Pediatric Hematology and Oncology
Hyogo	Hyogo Prefectural Kobe Children's Hospital	Department of Hematology and Oncology
Hyogo	Hyogo Prefectural Tamba Medical Center	Department of Hematology
Hyogo	Hyogo Prefectural Tamba Medical Center	Department of Internal Medicine
Hyogo	Japan Community Health Care Organization Kobe Central Hospital	Department of Internal Medicine
Hyogo	Kakogawa City Central Hospital	Department of Respiratory Medicine
Hyogo	Kansairosai Hospital	Department of Dermatology
Hyogo	Kawasaki hospital	Department of Hematology and Oncology
Hyogo	Kobe City Medical Center General Hospital	Department of General Pediatrics
Hyogo	Kobe University Hospital	Department of Pediatrics
Hyogo	Konan Medical Center	Department of Hematology and Oncology
Hyogo	Shinko Hospital	Department of Hematology
Hyogo	Takarazuka City Hospital	Department of Hematology
Nara	Kindai University Nara Hospital	Department of Hematology
Nara	Kindai University Nara Hospital	Department of Respiratory Medicine and Allergology
Nara	Minami-Nara General Medical Center	Department of Infectious Diseases
Nara	Nara Medical University Hospital	Department of Pediatrics
Nara	Nara Prefecture General Medical Center	Department of Infectious Diseases
Nara	Yamatotakada Municipal Hospital	Department of Dermatology
Wakayama	Japanese Red Cross Wakayama Medical Center	Department of Infectious Diseases
Wakayama	Wakayama Medical University Hospital	Department of Hematology and Oncology
Wakayama	Wakayama Medical University Hospital	Department of Infection Control and Prevention
Wakayama	Wakayama Medical University Hospital	Department of Pediatrics
Tottori	Tottori University Hospital	Department of Pediatrics
Shimane	National Hospital Organization Hamada Medical Center	Department of General Medicine
Shimane	Shimane Prefectural Central Hospital	Department of Infectious Diseases
Shimane	Shimane University Hospital	Department of Pediatrics
Okayama	Japanese Red Cross Okayama Hospital	Division of Hematology
Okayama	Kaneda Hospital	Department of Internal Medicine

Okayama	Kawasaki Medical School General Medical Center	Department of General Internal Medicine 1
Okayama	Kawasaki Medical School Hospital	Department of Respiratory Medicine
Okayama	Kawasaki Medical School Hospital	Department of Pediatrics
Okayama	Kurashiki Central Hospital	Department of Pediatrics
Okayama	Kurashikikinen Hospital	Department of Respiratory Medicine
Okayama	National Hospital Organization Okayama Medical Center	Division of Infectious Diseases
Okayama	Okayama City Hospital	Department of Clinical Laboratory
Okayama	Okayama Rosai Hospital	Department of Internal Medicine
Hiroshima	Hiroshima City Asa Hospital	Department of Hematology
Hiroshima	Hiroshima City Hospital	Department of Hematology
Hiroshima	Hiroshima University Hospital	Department of Infectious Diseases
Hiroshima	Hiroshima University Hospital	Department of Pediatrics
Hiroshima	National Hospital Organization Fukuyama Medical Center	Department of Infectious Diseases
Yamaguchi	JCHO Shimonoseki Medical Center	Department of Hematology
Yamaguchi	Shimonoseki City Hospital	Department of Respiratory Medicine
Tokushima	Anan Medical Center	Department of Internal Medicine
Tokushima	Tokushima University Hospital	Department of Hematology
Kagawa	Kagawa Prefectural Central Hospital	Department of Hematology
Kagawa	Kagawa University Hospital	Department of Respiratory Medicine
Kagawa	Takamatsu Municipal Hospital	Department of Surgery
Kagawa	Watanabe Iin	Department of Dermatology and Urology
Ehime	Ehime Prefectural Central Hospital	Department of Pediatrics
Ehime	Ehime University Hospital	Department of Hematology, Clinical Immunology and Infectious Diseases
Kochi	Kochi Medical School Hospital	Department of Hematology
Kochi	Kochi Medical School Hospital	Department of Respiratory Medicine and Allergology
Kochi	Kochi Red Cross Hospital	Department of Internal Medicine
Kochi	National Hospital Organization Kochi Hospital	Department of Internal Medicine
Fukuoka	Fukuoka Kinen Hospital	Department of Internal Medicine
Fukuoka	Fukuoka University Chikushi Hospital	Department of Respiratory Medicine
Fukuoka	Fukuoka University Hospital	Department of Respiratory Medicine
Fukuoka	Hospital of University of Occupational and Environmental Health	Department of Pediatrics
Fukuoka	Hospital of University of Occupational and Environmental Health	Department of Respiratory Medicine
Fukuoka	Japan Community Health Care Organization Kyushu Hospital	Department of Hematology and Oncology
Fukuoka	Japanese Red Cross Fukuoka Hospital	Division of Hematology and Oncology
Fukuoka	Kenwakai Otemachi Hospital	Division of Infectious Diseases
Fukuoka	Kitakyusyu City Yahata Hospital	Department of Pediatric Hematology and Oncology
Fukuoka	Kitakyusyu Municipal Medical Center	Department of Respiratory Medicine
Fukuoka	Kitakyusyu Municipal Medical Center	Division of Hematology
Fukuoka	Kurume University Hospital	Division of Infection Control and Prevention
Fukuoka	Kyushu Cancer Center	Department of Hematology and Cell Therapy
Fukuoka	Kyushu Cancer Center	Department of Pediatrics
Fukuoka	Kyushu Rosai Hospital	Department of Internal Medicine
Fukuoka	Kyushu university hospital	Department of Clinical Immunology, Rheumatology & Infectious Disease
Fukuoka	National Hospital Organization Fukuoka higashi medical center	Department of Hematology
Fukuoka	National Hospital Organization Fukuoka National Hospital	Department of Internal Medicine
Fukuoka	National Hospital Organization Kyushu Medical Center	Department of Infectious Diseases
Fukuoka	Shin-kokura Hospital	Department of Respiratory Medicine
Fukuoka	Steel Memorial Yawata Hospital	Department of Dermatology
Fukuoka	Wakamatsu Hospital of the University of Occupational and Environmental Health	Department of Palliative Care and Hematology and Oncology
Saga	Saga University Hospital	Department of Infectious Disease and Hospital Epidemiology
Nagasaki	Inoue Hospital	Department of Internal Medicine
Nagasaki	Japanese Red Cross Nagasaki Genbaku Hospital	Department of Respiratory Medicine
Nagasaki	Japanese Red Cross Nagasaki Genbaku Isahaya Hospital	Department of Respiratory Medicine
Nagasaki	Nagasaki Harbor Medical Center	Department of Respiratory Medicine
Nagasaki	Nagasaki University Hospital	Infection Control and Education Center
Nagasaki	Sasebo Chuo Hospital	Department of Respiratory Medicine
Nagasaki	St. Francis Hospital	Department of Internal Medicine and Hematology
Kumamoto	Japan Community Health Care Organization Kumamoto General Hospital	Department of Hematology
Kumamoto	Kumamoto Chuo Hospital	Department of Respiratory Medicine
Kumamoto	Kumamoto City Hospital	Department of Hematology and Oncology
Kumamoto	Kumamoto City Hospital	Division of Infectious Diseases
Kumamoto	Kumamoto Shinto General Hospital	Department of Hematology
Kumamoto	Kumamoto University Hospital	Department of hematology
Kumamoto	NHO Kumamoto-minami Hospital	Department of Internal Medicine
Kumamoto	Saiseikai Kumamoto Hospital	Division of Respiratory Medicine
Oita	Nagato Memorial Hospita	Department of Internal Medicine
Oita	Oita Prefectural Hospital	Department of Hematology
Oita	Oita Prefectural Hospital	Division of Infectious Diseases
Oita	Oita Prefecture Saiseikai Hita Hospital	Department of Hematology
Oita	Oita University Hospital	Department of Hematology

Miyazaki	Miyazaki Prefectural Miyazaki Hospital	Department of Internal Medicine
Miyazaki	Miyazaki Prefectural Miyazaki Hospital	Department of Pediatrics
Miyazaki	Miyazaki-ken Saiseikai Hyuga Hospital	Department of Internal Medicine
Miyazaki	National Hospital Organization Miyazaki Higashi Hospital	Department of Respiratory Medicine
Miyazaki	University of Miyazaki Hospital	Department of Infectious Diseases
Kagoshima	Ikeda hospital	Department of Hematology
Kagoshima	Kagoshima City Hospital	Division of internal medicine
Kagoshima	Kagoshima University Hospital	Department of Pediatrics
Kagoshima	National Hospital Organization Kagoshima Medical Center	Department of Pediatrics
Okinawa	Nakagami Hospital	Department of Infectious Diseases
Okinawa	Okinawa Chubu Hospital	Division of Infectious Diseases
Okinawa	Okinawa Prefectural Nanbu Medical Center and Children's Medical Center	Department of Hematology and Oncology
Okinawa	University of the Ryukyus Hospital	Department of Dermatology

Names of regions in Japan are arranged in order from north to south in this table. Institution and department names are listed in alphabetical order.
